# Molecular and chemical dialogues in **bacteria-protozoa interactions**

**DOI:** 10.1038/srep12837

**Published:** 2015-08-06

**Authors:** Chunxu Song, Mark Mazzola, Xu Cheng, Janina Oetjen, Theodore Alexandrov, Pieter Dorrestein, Jeramie Watrous, Menno van der Voort, Jos M. Raaijmakers

**Affiliations:** 1 Laboratory of Phytopathology, Wageningen University, 6708 PB Wageningen, the Netherlands; 2 USDA-ARS, 1104 N. Western Ave., Wenatchee, Washington 98801; 3 MALDI Imaging Lab, University of Bremen, 28359 Bremen, Germany; 4 Center for Industrial Mathematics, University of Bremen, 28359 Bremen, Germany; 5 SCiLS GmbH, 28359 Bremen, Germany; 6 Skaggs School of Pharmacy and Pharmaceutical Sciences, University of California at San Diego , San Diego, California 92093, United States; 7 Structural and Computational Biology Unit, European Molecular Biology Laboratory (EMBL), 69117 Heidelberg, Germany; 8 Microbial Ecology Department, Netherlands Institute of Ecology (NIOO-KNAW), 6708 PB Wageningen, the Netherlands

## Abstract

Protozoan predation of bacteria can significantly affect soil microbial community composition and ecosystem functioning. Bacteria possess diverse defense strategies to resist or evade protozoan predation. For soil-dwelling *Pseudomonas* species, several secondary metabolites were proposed to provide protection against different protozoan genera. By combining whole-genome transcriptome analyses with (live) imaging mass spectrometry (IMS), we observed multiple changes in the molecular and chemical dialogues between *Pseudomonas fluorescens* and the protist *Naegleria americana*. Lipopeptide (LP) biosynthesis was induced in *Pseudomonas* upon protozoan grazing and LP accumulation transitioned from homogeneous distributions across bacterial colonies to site-specific accumulation at the bacteria-protist interface. Also putrescine biosynthesis was upregulated in *P. fluorescens* upon predation. We demonstrated that putrescine induces protozoan trophozoite encystment and adversely affects cyst viability. This multifaceted study provides new insights in common and strain-specific responses in bacteria-protozoa interactions, including responses that contribute to bacterial survival in highly competitive soil and rhizosphere environments.

The rhizosphere is home to diverse organisms including bacteria, fungi, oomycetes, nematodes, protozoa, algae, viruses, archaea and arthropods^1^,^2^,^3^,^4^. Elevated densities of microorganisms in the rhizosphere lead to concomitant increases in the populations and feeding activities of their predators^5^. Bacteriovorous free-living nematodes and protozoa can have a significant impact on the bacterial community composition in soil and rhizosphere environments^6^,^7^. Preferential feeding has been reported for protozoans in soil ecosystems with bacterial genera such as *Pseudomonas* being favored over other genera such as *Streptomyces* and *Bacillus*^8^,^9^. In turn, bacteria possess multiple defense strategies to resist or evade predation by protozoa via both intracellular and extracellular adaptations^10^. Intracellular adaptations include survival and replication of bacteria inside the protozoan cell^11^. Extracellular avoidance mechanisms include altered cell morphology^12^,^13^,^14^, increased bacterial motility^15^, biofilm formation^16^ and production of bioactive compounds^16^,^17^,^18^.

For plant-associated *Pseudomonas* species, hydrogen cyanide (HCN), 2,4-diacetylphloroglucinol (2,4-DAPG) and pyrrolnitrin (PRN) were shown to contribute to defense against protozoa^19^,^20^. Also extracellular proteases inhibit protozoan predation in *Pseudomonas*^21^ as well as in *Vibrio cholerae*^22^,^23^. We previously assessed the function of lipopeptide surfactants (LPs) as a bacterial defense mechanism against protozoan predation: LPs were shown to limit protozoan grazing of *Pseudomonas fluorescens* both *in vitro* and *in situ*^24^. Interestingly, protozoa-*Pseudomonas* interactions led to enhanced transcription of LP biosynthesis genes^24^. These results suggested that bacteria can modulate the production of secondary metabolites in response to protozoan predators. However, evidence that LPs are actually produced during protozoa-*Pseudomonas* interactions is lacking. Also knowledge of the overall chemistry and transcriptional responses at the bacteria-protozoa interaction site remain elusive. The aim of this study was to unravel predation-mediated responses at the interface of *Pseudomonas*-protozoa interactions. To that end, we conducted whole-genome transcriptome, MALDI-TOF-based imaging mass spectrometry (IMS)^25^,^26^,^27^ and live colony NanoDESI mass spectrometry to monitor, *in situ*, changes in gene expression and production of metabolites during bacteria-protozoa interactions.

## Results and Discussion

### Transcriptional response of ***P. fluorescens*** SS101 - protozoa interactions

**Figure 1:**
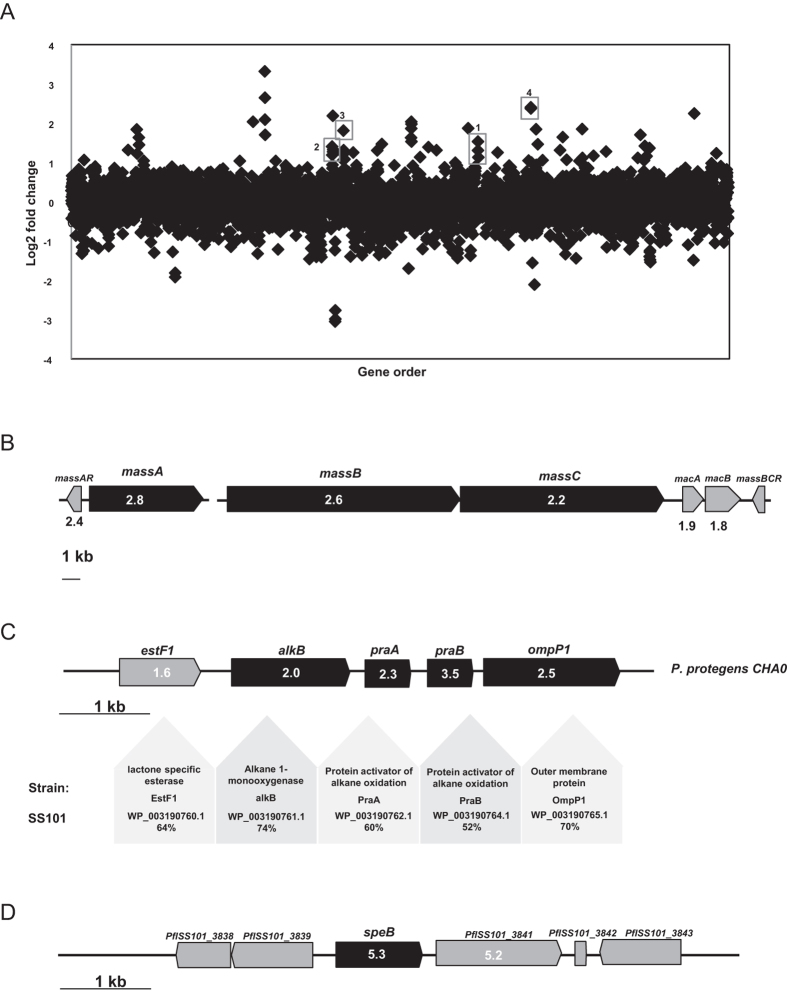
(**A**) Transcriptomic analysis of *P. fluorescens* SS101-*N. americana* interaction. Each point represents one annotated gene in the SS101 genome, with the X-axis showing the gene order, and the Y-axis showing the log2 of gene transcript abundance in the interaction. The identities of highly modulated, well-characterized gene clusters are shown. 1. *massA*; 2. *massB*, *massC*; 3. alkane oxidation gene clusters; 4. agmatinase encoding gene *speB*. (**B**) Organization of the lipopeptide (LP) gene cluster in *P. fluorescens* SS101. The three LP biosynthesis genes are designated *massA*, *massB* and *massC*. In the boxes of the genes are the fold changes in their expression during *P. fluorescens-N. americana* interaction. (**C**) Organization of the alkane oxidation gene cluster in SS101. The reference strain used is *P. protegens* CHA0 (previously described as *P. fluorescens*). In the boxes of the genes are the fold changes in their expression during *P. fluorescens-N. americana* interaction. (**D**) Organization of the putrescine encoding gene *speB* and its flanking genes. In the boxes of the genes are the fold changes in their expression during *P. fluorescens-N. americana* interaction.

When challenged with *N. americana,* up to 2.3% of the SS101 genes exhibited significantly altered expression in cells located at the interaction interface. In total, 128 genes were differentially expressed in SS101 with 55 genes up-regulated and 73 genes down-regulated (fold-change >2.0; P value < 0.05) (Fig. 1A). The LP biosynthesis genes *massA*, *massB* and *massC* in SS101 were more than 2-fold up-regulated (Fig. 1B). This up-regulation is consistent with qRT-PCR results obtained previously^24^. Also the massetolide-specific *luxR*-type transcriptional regulatory gene *massAR* and the downstream ABC-type efflux genes *macA* and *macB* were significantly up-regulated (Fig. 1B). Several of the other differentially regulated genes (17 and 29 genes up and down, respectively) were classified as “Function unknown” or “Not in COGs” categories (Figure S1, category S and X, respectively). These results suggest that a large proportion of the bacterial genes expressed in response to *N. americana* are unknown and remain to be characterized. Thirteen out of sixteen genes from the “Amino acid transport and metabolism” category were up-regulated, including genes associated with arginine and proline metabolism, lysine biosynthesis, degradation of aromatic compounds and phenylalanine metabolism, respectively. The *yveA* gene, which mediates uptake of both l-aspartate and l-glutamate^28^, was 3-fold up-regulated in SS101 in interaction with *N. americana* (Table S1). PflSS101_1522, a homologue gene of *ilvB* in *Pseudomonas protegens*, was 4-fold up-regulated in SS101 upon interaction with *N. americana*. IlvB is a large subunit of acetohydroxyacid synthase (AHAS) which catalyses the first step in the biosynthesis of the essential amino acids isoleucine, leucine and valine in bacteria, as well as in plants, fungi and certain algae^29^,^30^. The up-regulation of several genes involved in amino acid transport and metabolism suggests that the interaction with *N. americana* induces changes in primary metabolism of *P. fluorescens* SS101. In our previous study^31^, we found indications that amino acids affect the production of the lipopeptide massetolide A in SS101. Hence, the observed transcriptional changes in amino acid metabolism may, via LP biosynthesis, modulate defense against protozoan predation.

In SS101, the extracellular alkaline metalloprotease encoding gene *aprA* was 2.4-fold up-regulated. Although the role of AprA in defense of SS101 against protozoan predation remains to be tested, proteases are known to contribute to the defense of *P. protegens* CHA0, Pf-5 and *Vibrio cholerae* to protozoa^21^,^22^,^23^,^32^. Among the down-regulated genes were 5 genes from the role category “inorganic ion transport and metabolism” (Figure S1). Another down-regulated gene was a TetR family transcriptional regulator (PflSS101_2501). The TetR-family of transcriptional regulators (TFR) is a large and important family of one-component signal transduction systems^33^. TFRs are known to interact with an exceptionally diverse set of small molecules, including antibiotics, metabolites, and cell-cell signalling molecules^34^. For instance, the macrolide antibiotic, avermectin, produced by *S. avermitilis*, was recently shown to be negatively regulated by a TetR-family transcriptional regulator^35^. The function of the TFR genes in interactions between *P. fluorescens* SS101 and *N. americana* remains unknown.

### Alkane oxidation/degradation genes up-regulated in bacteria - protozoa interactions

In the SS101-protozoa interaction, we observed that the gene cluster PflSS101_2280–2283 was up-regulated, with significant fold changes ranging from 2.0 to 3.5 (Fig. 1C). BlastX analysis revealed that these genes are orthologues (52%–74% identities) of *alkB*, *praA*, *praB* and *ompP1* of the alkane oxidation/degradation gene cluster from *P. protegens* CHA0 (Fig. 1C). AlkB encodes an integral membrane alkane hydroxylase which is essential for growth of *P. protegens* CHA0 on C12-C16 n-alkanes. Inactivation of this gene significantly reduced the capacity of CHA0 to protect plants against soil-borne diseases such as black root rot of tobacco and take-all disease of wheat (Smits, 2001). PraA and PraB are two activators of alkane oxidation and showed alkane-solubilizing effects after overexpression in *E. coli*. A *praA* mutant in *P. aeruginosa* PG201 was found to be retarded in its growth in n-hexadecane-containing media^36^. Additionally, genes involved in the alkane degradation process including alcohol dehydrogenase (PflSS101_1413, *adhB*) and aldehyde dehydrogenase (PflSS101_2843) were up-regulated in the *Pseudomonas*-protozoa interactions. Some *Pseudomonas* species employ biosurfactant-mediated solubilisation to enable use of long chain alkanes as a carbon source^37^,^38^. This process may function as a means to store excess carbon which can subsequently be utilized by the bacterium as an endogenous energy source during starvation periods^39^. Alternatively, products of the alkane oxidation could serve as precursors for the production of certain antifungal secondary metabolites, such as 2,4-DAPG^40^,^41^. Examination of such a premise and the potential link between alkane degradation and lipopeptide biosynthesis in strain SS101 is not known but would be interesting to examine in future studies.

### A putrescine catalysis encoding gene is up-regulated in bacteria - protozoa interactions

We observed that the agmatinase encoding gene *speB* (PflSS101_3840) was more than 5-fold up-regulated in the SS101-protozoa interaction (Fig. 1D). In bacteria, the gene product of *speB* is responsible for catalysing the conversion of agmatine to putrescine^42^,^43^. A transporter gene (PflSS101_3841), located adjacent to the up-regulated agmatinase gene was also up-regulated 5.2-fold in SS101 cells interacting with *N. americana* (Fig. 1D). Putrescine is a polyamine known to be involved in a variety of functions. It can be utilized by bacteria as both carbon and nitrogen source and is required for optimal growth^44^ and root colonization^45^. Putrescine can act as an intercellular signal for swarming in *Proteus mirabilis*^46^ and protects *Escherichia coli* cells from the toxic effects of oxygen^47^. It can also restore biofilm formation of an arginine decarboxylase (SpeA) and ornithine decarboxylase (SpeC) double mutant in *Yersinia pestis*^48^. These findings suggest that putrescine may provide protection, directly or indirectly, to strain SS101 against predation by *N. americana*.

### Effects of putrescine on ***N. americana*** viability

**Figure 2:**
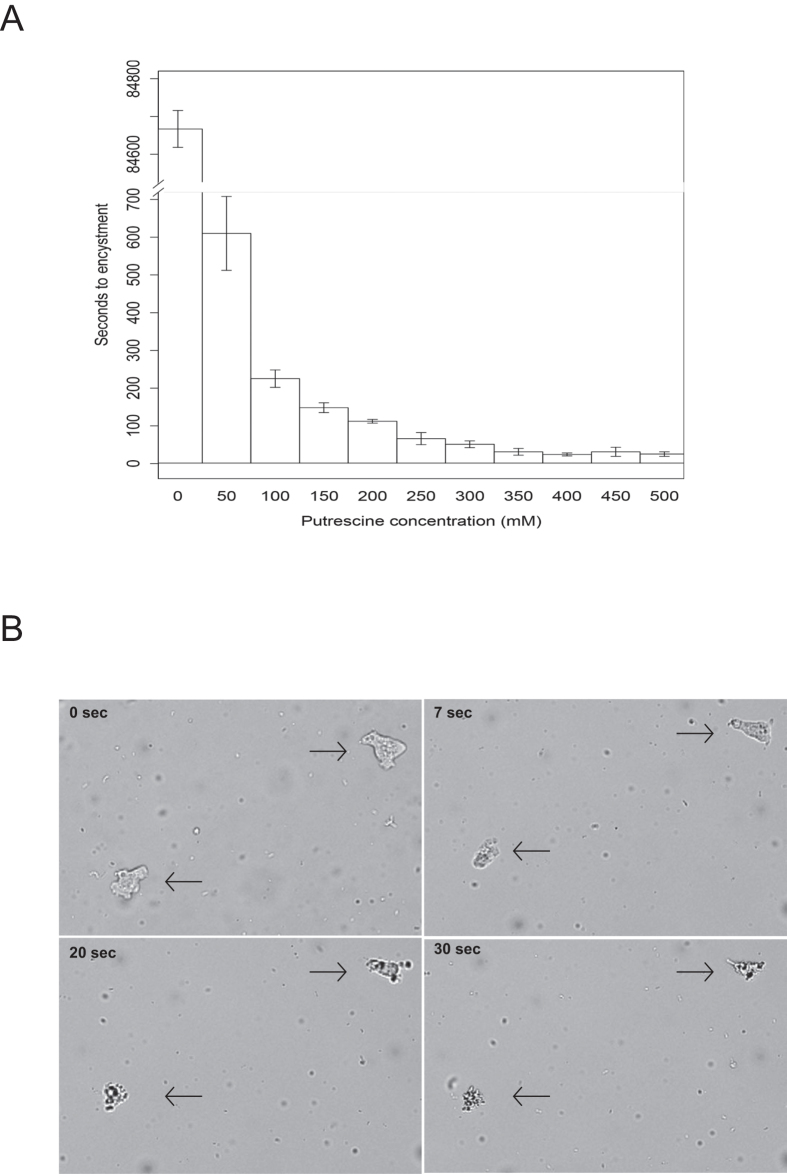
(**A**) Time to encystment of amoeboid and flagellate forms of *N. americana* exposed to increasing concentrations of putrescine. For each putrescine concentration, the average of three replicates is shown. Error bars refer to the standard error of the mean. (**B**) Representative images of trophozoite viability at 0, 7, 20 and 30 seconds after exposure to 250 mM putrescine. A replicate consisted of an individual sample containing the protist incubated in the indicated putrescine concentration from which a 6 μl sample was extracted and all cysts or trophozoites in that sample were enumerated.

*In vitro* assays were conducted to examine the effect of putrescine on trophozoites of *N. americana*. The results of dose-dependent experiments showed that putrescine induced trophozoite encystment (Fig. 2A). The time required for induction of trophozoite encystment decreased with increasing putrescine concentrations. At a putrescine concentration of 50 mM, all trophozoites encysted within approximately 10 min whereas at a concentration of 250 mM or higher, the time to encystment was approximately 1 min. From a concentration of 350 mM onward, there were no observable cysts. This was likely due to trophozoite lysis, which in some instances left visible remnants of deflated trophozoites. Already after 7 seconds exposure to 250 mM putrescine, trophozoites started to deflate (Fig. 2B). Subsequently, cyst viability was assessed by determining the average number of trophozoites obtained from putrescine-treated cysts transferred to the surface of water agar plates amended with heat-killed *E. coli*. Cyst viability decreased with prior exposure to increasing concentrations of putrescine (Table 1). Whether the putrescine concentrations used here are representative of the concentrations produced by the bacterium *in situ* remains to be determined.

### Metabolites produced in ***Pseudomonas***-protozoa interactions

**Figure 3:**
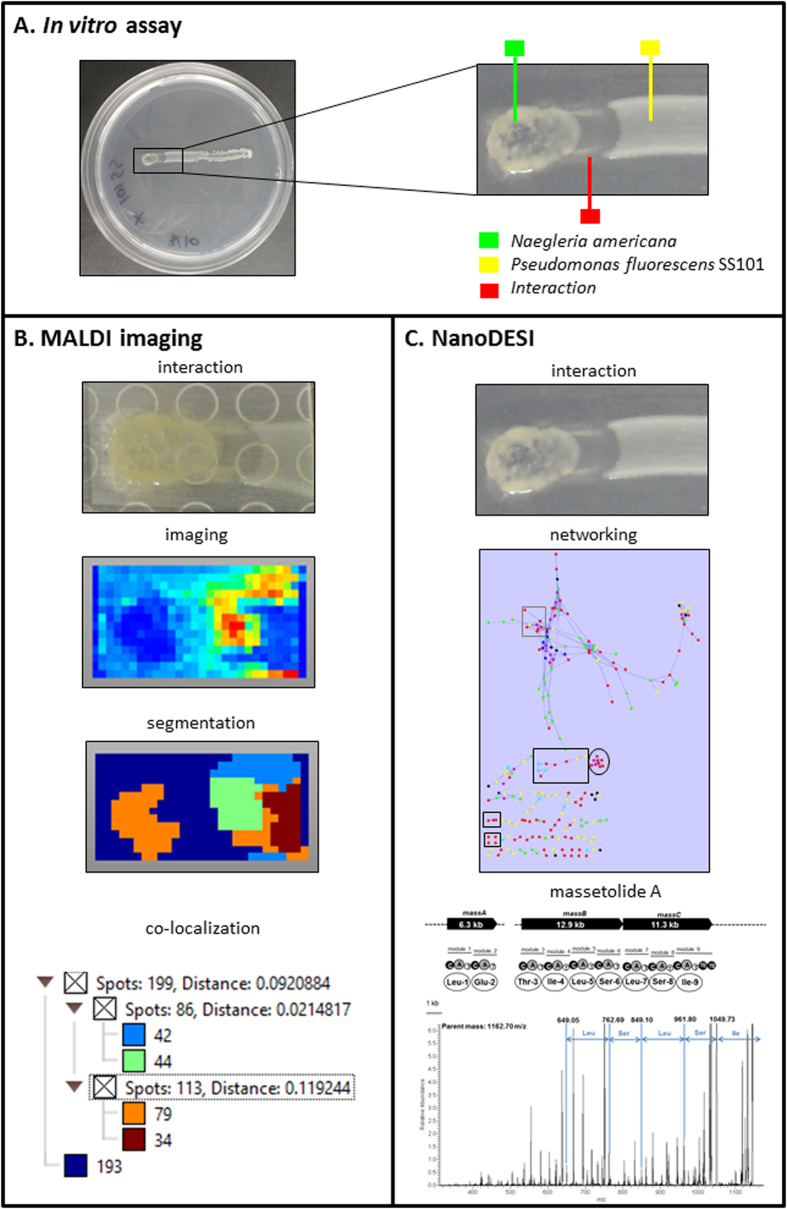
(**A**) Experimental setup to study *Pseudomonas*-protozoa interactions by MALDI imaging mass spectrometry (IMS). The green box-line indicates the protozoan predator *N. americana* alone; the red box-line indicates the interface of *P. fluorescens* SS101-*N. americana*; the yellow box-line indicates *P. fluorescens* SS101 alone. (**B**) MALDI-IMS analysis of the *Pseudomonas*-protozoa interaction, including imaging of metabolite classes, spatial segmentation and co-localization of the MALDI IMS data. (**C**) MS/MS network analysis and annotation of ion clusters from the *P. fluorescens* SS101-*N. americana* interaction. Ion clusters in the black squares represent the lipopeptide massetolide A and its derivatives; the black circle represents the 325-477 m/z ion cluster; the grey square represents the 766-796 m/z ion cluster. MS/MS analysis further indicated that the parent ion with 1162.70 m/z detected in the *P. fluorescens SS101*- *N. americana* interaction is most likely massetolide A. Complete lists of the ion clusters detected in the network analysis are given in Tables S4, S5 and S6.

In order to explore and identify specific classes of metabolites produced at the bacteria-protozoa interface, an experiment similar to that used for the transcriptional profiling was conducted. In brief, strain SS101 was streaked across the surface of solid 0.2 X NBY medium using an inoculation loop (Fig. 3A). After 3 h incubation at 25 °C, 5 μL of a suspension containing 200 *N. americana* cysts μL^−1^ was spotted at one end of the linear bacterial growth, and the plates were incubated at 25 °C for 3 days. A section of the agar containing the interaction zone was applied to MALDI-TOF to study the secreted metabolites by IMS (Fig. 3B). In addition, live colony NanoDESI mass spectrometry was performed on the protozoan colony, the interaction zone and the *Pseudomonas* colony to construct MS/MS metabolite networks. Nodes with a high MS/MS spectral analogy cluster together and often belong to the same chemical class^49^. Clusters of the different metabolite classes were then compared to the ions observed in the MALDI IMS data. We detected metabolites produced by *Pseudomonas* alone (yellow nodes), protozoa alone (green nodes) and produced during the *Pseudomonas-*protozoa interaction (red nodes). The network was constructed combing the different samples per species together (Fig. 3C).

### Metabolite classes in ***P. fluorescens*** SS101 - ***N. americana*** interaction

**Figure 4:**
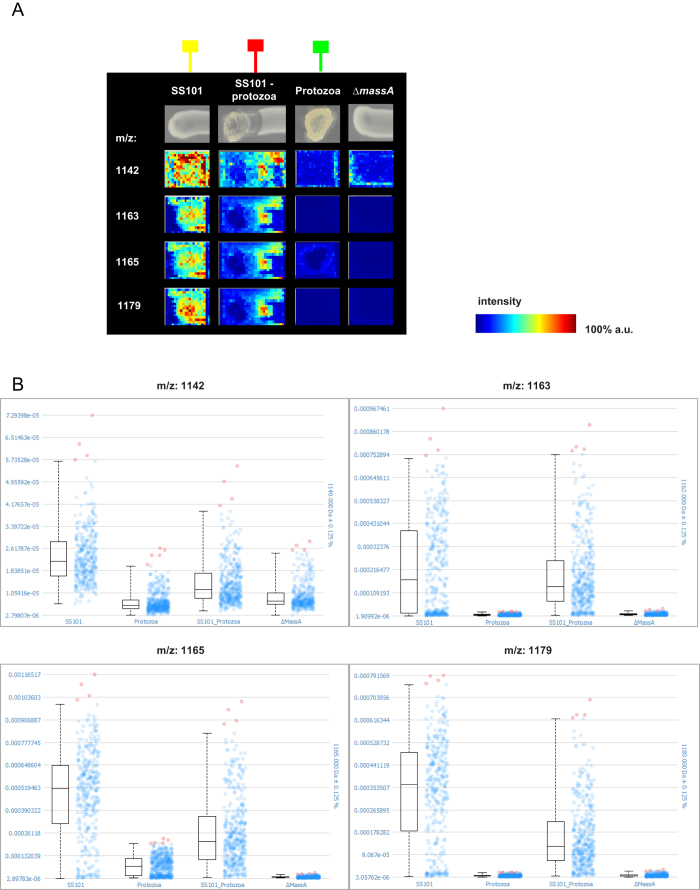
(**A**) MALDI imaging mass spectrometry (IMS) shows production of massetolide A and its derivatives during the *P. fluorescens* SS101-*N. americana* interaction. a.u. = arbitrary units. (**B**) Box plots depicting the production of massetolide A and its derivatives (m/z 1142, 1163, 1165 and 1179) in *P. fluorescens* SS101 alone (SS101), *N. americana* alone (Protozoa), *P. fluorescens* SS101-*N. americana* interaction (SS101_Protozoa) and *massA* mutant alone (Δ*massA*). (**B**) The box plots represent the median intensity in arbitrary units after TIC normalization (horizontal line), the upper and lower quartiles (box layout, spectra in which the intensities are within a range of 25%–75% of the data), the upper and lower quantiles (dashed lines, spectra in which the intensities are within a range of 0%–99%) as well as the outliers (spectra with intensities greater than 99% of the data). The green box-line indicates *N. americana* alone; the red box-line indicates *P. fluorescens* SS101-*N. americana* interaction; the yellow box-line indicates *P. fluorescens* SS101 alone.

Spatial segmentation analysis of the MALDI IMS data revealed four specific classes of metabolites indicated with different colours (light blue, green, orange and dark red), that were co-localized in the *Pseudomonas*-protozoa interaction zone (Fig. 3B; Table S2). There were 6 and 14 ions found to be co-localized within the light blue and green cluster, respectively, with correlation values greater than 0.5 (Fig. 3B; Table S2) including 8 ions with predicted masses ranging from 1136 m/z to 1201 m/z (Fig. 4A). These ions were not detected in the massetolide-deficient ∆*massA* mutant or *N. americana* alone (Fig. 4A). The absence of these ions in the ∆*massA* mutant suggests that they are massetolide derivatives. Box plots further confirmed that the intensities of these ions were higher in wild type strain SS101, and in the SS101-protozoa interaction than in the samples with *N. americana* or ∆*massA* alone (Fig. 4B). Masses of massetolide A and its derivatives also clustered together in the MS/MS network (Fig. 3C; Table S4). Tandem MS of the ion with a mass of 1163 m/z indicated a peptide sequence of leucine, serine, leucine, serine and isoleucine. These amino acids are identical to the C-terminal peptide sequence of massetolide A (Fig. 3C). Based on our previous study, the mass of massetolide A is 1140 and the masses of its derivatives range from 1112–1158 m/z^50^. The larger ion masses detected here are most likely due to a sodium (molecular weight: 22.989) gain during ionization. Although the intensities of the massetolides were not different between SS101 and SS101-protozoa interaction (Fig. 4B), we observed a striking difference in spatial distribution of massetolide A. In absence of the protozoa, the lipopeptide was more homogeneously distributed in the SS101 colony, whereas in presence of protozoa it localized predominantly in/around the bacterial cells at the interaction zone (Fig. 4A). This result was reinforced by the absence of massetolides in the interaction between the *massA* mutant and the protozoan predator (Figure S2). To our knowledge, this is the first report of the real time visualization and spatial distribution of LPs during bacteria-protozoa interactions.

**Figure 5:**
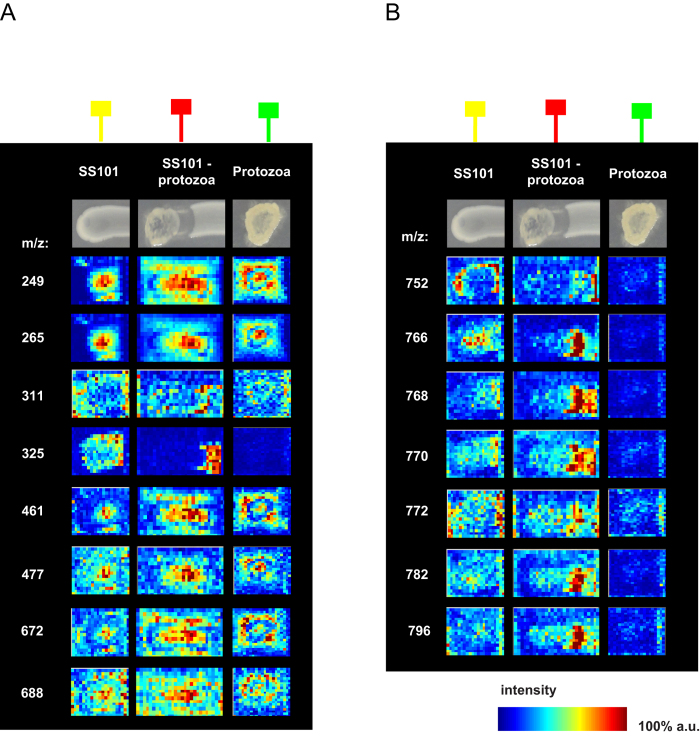
(**A**) MALDI imaging mass spectrometry (IMS) shows production of 249–688 m/z ions in the MS/MS network of the *P. fluorescens* SS101-*N. americana* interaction. a.u. = arbitrary units. (**B**) MALDI imaging mass spectrometry (IMS) shows production of 752–796 m/z ions cluster in the MS/MS network of the *P. fluorescens* SS101-*N. americana* interaction. The green box-line indicates *N. americana* alone; the red box-line indicates *P. fluorescens* SS101-*N. americana* interaction; the yellow box-line indicates *P. fluorescens* SS101 alone.

Apart from the massetolides, other ions with predicted masses ranging from 249–688 m/z were co-localized with the green cluster in the segmentation map (Fig. 3B, Table S2). These ions were detected in the interaction, in bacteria or in the protozoa alone (Fig. 5A; Table S2). One of the ions with a mass of 477 m/z clustered with seven other ions in the MS/MS network (Fig. 3C; Table S5). Ion 325 m/z, whose intensity is higher in the bacteria-protozoa interaction than in the bacteria alone (Fig. 5A), will need to be investigated in more detail by tandem MS analyses to resolve its identity. Furthermore, next to the representative ions shown in Fig. 5B, a number of other ions were present (Table S2) including three ions with a mass of 740 m/z, 741 m/z and 767 m/z belonging to the green class, and sixteen ions with masses ranging from 703 m/z to 790 m/z (Table S2) belonging to the dark red class from the segmentation map. The ion with a mass of 766 m/z clustered in the network with nine other ions with masses ranging from 752 m/z to 796 m/z (Fig. 3C; Table S6). Preliminary MS/MS analyses showed that four of these ions exhibit a similar fragmentation pattern with a 123.9 Da loss (not shown). Resolving the identity of this metabolite class will be subject of future experiments.

### General and strain-specific responses to protozoan predation

To determine if the observed transcriptional and metabolic responses are specific for strain SS101 or more generally found in *P. fluorescens*, we conducted similar studies employing *P. fluorescens* SBW25. At the transcriptional level, 135 genes showed differential expression in SBW25, with 65 genes up-regulated and 70 genes down-regulated (fold-change >2.0; *P* value < 0.05). Twenty five genes in SBW25 exposed to protozoa showed a similar transcriptional response as their orthologues in strain SS101 (Figure S3, Table S3). The 17 up-regulated genes included the viscosin biosynthesis genes, the alkane degradation gene cluster and also *speB*, the agmatinase gene involved in putrescine biosynthesis. At the metabolic level, viscosin production in SBW25-protozoa interaction was confirmed (Figure S4,S5). Similar to that observed for massetolide produced by SS101, also the lipopeptide viscosin was localized at the interaction site between SBW25 and *N. americana*, whereas in the absence of protozoa the viscosin was more homogeneously distributed in the bacterial colony. This indicates that lipopeptide accumulation at the bacteria-protozoa interface constitutes a defense mechanism for at least two *Pseudomonas fluorescens* strains when confronted with protozoa.

A specific ion was detected with a mass of 88.66 and 88.348 m/z by MALDI IMS for SS101 and SBW25 respectively (Figure S6). The theoretical mass of protonated putrescine ion species is [M + H]^+^89.1 amu. These data suggest that the detected ion is putrescine, further supporting the transcriptome data for *P. fluorescens* SS101 and SBW25 (Figure S3). Similar to SS101, production of ions with masses of 325 m/z and 766 m/z were detected in the SBW25-protozoa interaction (Figure S7). Comparisons of the MS/MS profiles with the ones found in SS101-protozoa interaction indicated that these represent the same metabolite class(es) produced in the interactions for both strains. Strain-specific metabolites were also observed in the SBW25-protozoa interaction (Figure S8,S9) and the identities of these metabolites are currently under investigation.

## Conclusions

Whole genome transcriptomic analysis of *Pseudomonas fluorescens* SS101 in confrontation with the protozoan predator *N. americana* revealed altered expression for 2.3% of genes from the SS101 genome. Among these changes, lipopeptide biosynthesis genes, together with the adjacent transcriptional regulator were up-regulated, which extended our initial findings of the role of lipopeptides as an anti-predation defense mechanism^24^. Moreover, we showed that putrescine biosynthesis in SS101 was up-regulated in response to challenge by *N. americana*. Subsequent experiments revealed, for the first time, that putrescine induces protozoan trophozoite encystment and affects cyst viability. Subsequent to the transcriptomic analysis, metabolic analysis of this interaction was conducted via MALDI imaging mass spectrometry (IMS) and live colony NanoDESI mass spectrometry. To date, most information on these techniques focuses on microbes alone^49^, bacteria-bacteria or bacteria-fungi interactions^51^,^52^,^53^. Here, new information is provided on the chemistry of bacteria-protozoa interactions. Our study identified, for the first time, real time and site-specific lipopeptide production at the interface of *Pseudomonas*-protozoa interactions and demonstrated that closely related bacterial strains exhibit common and unique transcriptomic and metabolic responses to predation.

## Material and Methods

### Protozoa, bacteria and growth conditions

*Pseudomonas fluorescens* strains SS101 and SBW25 were grown on Pseudomonas agar F (Difco) plates or in liquid King’s medium B (KB) at 25 °C. *Escherichia coli* was grown on Luria-Bertani (LB) plates or in LB broth. The amoeba-flagellate *Naegleria americana* was used as the protozoan predator. The protist was propagated by cultivation with heat-killed *E. coli* DH5α as the food source. 5 μL of bacterial cells (∼10^8^ cells) were added to a water agar surface contained in a 9-cm-diameter petri plate, and was subsequently overlaid with 3 ml of Page’s modified Neff’s amoeba saline (PAS) solution^54^. The plates were then inoculated with 200 μl of a *N. americana* cyst suspension (200 cysts μl^−1^), sealed with Parafilm, and incubated at 20°C with 2 ml PAS added to the plates at 7-day intervals.

### *Pseudomonas-N. americana* interaction assay

Bacterial strains pre-cultured on KB agar were streaked across the surface of 0.2 X nutrient broth-yeast extract (NBY) (1 Liter contains 1.6 g nutrient broth, 0.4 g yeast extract, 1.0 g glucose, 15 g agar) at a width of 4 mm using a transfer loop. After 3 hours incubation at 25 °C, 5 μl of a suspension containing 200 *N. americana* cysts μl^−1^ was spot-inoculated at one end of the linear bacterial growth, and the plates were incubated at 25 °C for 3 days. Bacterial cells were collected from the zone of interaction using a transfer loop with 3 replicates for each strain.

### The effect of putrescine on encystment and viability of *N. americana*

Putrescine was added to an aqueous environment to *N. americana* trophozoites to final concentrations of 50–500 mM. Encystment of the trophozoites when exposed to putrescine was determined microscopically. For each putrescine concentration, four replicates were used.

To determine viability of the *N. americana* cysts, putrescine-treated cysts were transferred to water agar plates with PAS and heat-killed *E. coli*. The average number of trophozoites emerging from the cysts was determined microscopically after 24 hrs of incubation at 24 °C. Microscopic photos with 100X magnification were taken after 0, 7, 20 and 30 seconds of exposure to 250 mM putrescine using a Zeiss confocal microscope with transmitted light.

### Transcriptional profiling

*Pseudomonas fluorescens* strains SS101 and SBW25 were collected from the bacteria-protozoa zone of interaction and at the corresponding location on the control plates not inoculated with *N. americana*. Total RNA was extracted from the bacterial cells with Trizol reagent (Invitrogen) and further purified with the NucleoSpin RNA kit. Four replicates were used for each bacterial strain. Tiling microarrays for *Pseudomonas fluorescens* SS101 and SBW25 were developed in the Dutch Genomics Service & Support Provider, University of Amsterdam (UvA), Amsterdam, the Netherlands. In total, 134,276/134,858 probes (60-mer) were designed with, in general, a gap of 32/46 nucleotides between adjacent probes on the same strand and an overlap by 14/7 nucleotides when regarding both strands in SS101 and SBW25, respectively. In addition, 5,000 custom negative control probes were hybridized and used as an internal control to validate and normalize the designed probes in a CGH experiment of 4 arrays. Probes were annotated and assembled into probe sets for known genes based on location information retrieved from the Pathosystems Resource Integration Center (PATRIC, http://patricbrc.org). Probes outside of known genes were labelled as InterGenic Region (IGR). cDNA labelling was conducted as described previously^55^. Briefly, cDNA was synthesized in the presence of Cy3-dUTP (Cy3) for the test samples and with Cy5-dUTP (Cy5) for the common reference. The common reference was made by an equimolar pool of the test samples (3 μg per sample). 5 μg of total RNA per reaction was used and yielded 1.5–2.5 μg cDNA for each sample with larger than 16 pmol of Cy3 or Cy5 dye per microgram.

Hybridizations were performed according to Pennings *et al.*^56^. Slides were washed according to the procedures described in the Nimblegen Arrays User’s Guide - Gene Expression Arrays Version 5.0 and scanned in an ozone-free room with an Agilent DNA microarray scanner G2565CA (Agilent Technologies). Feature extraction was performed with NimbleScan v2.5 (Roche Nimblegen). Data pre-processing consisted of log_2_-transformation of the raw probe-intensity data, followed by a within slide Lowess normalization. Thus normalized sample (Cy3) channel intensities were summarized into probe set values and normalized between arrays using the RMA (Robust Multi-Array Analysis) algorithm (Irizarry, *et al.* 2003). All results described were found to be significant using a false discovery rate of less than 5%. Analysis of the gene expression data was conducted by Arraystar software. Microarray data were validated by quantitative PCR experiments for several genes (data not shown).

### MALDI-imaging mass spectrometry (IMS) and live colony mass spectrometry (NanoDESI) of *Pseudomonas*-*N. americana* interactions

*Pseudomonas* strains SS101, SBW25 and their lipopeptide deficient *massA* and *viscA* mutants, respectively, were streaked across the surface of solid 0.2 X NBY medium at a length of 4-cm using an inoculation loop. After 3 h incubation at 25 °C, 5 μL of a suspension containing 200 *N. americana* cysts μL^−1^ was spotted at one end of the linear bacterial growth, and the plates were incubated at 25 °C for 3 days. Thin layer interaction agar plates of *Pseudomonas* and *N. americana* were prepared and then sprayed with Universal MALDI matrix (Sigma-Aldrich). MALDI-imaging of the interaction samples on a Bruker MSP 96 anchor plate was performed on a Microflex Bruker Daltonics mass spectrometer outfitted with Compass 1.2 software suite^49^. To detect metabolites produced in the interaction zone, *Pseudomonas*-*N. americana* interaction plates were used to perform live colony mass spectrometry using NanoDESI as described previously^49^.

### SCiLS Lab analysis of MALDI-imaging mass spectrometry (IMS) data

The software SCiLS Lab version 2014b (SCiLS, Bremen, Germany) was used for MALDI-IMS data analysis to detect ions that have higher abundance at a specific condition, i.e. the bacteria-protozoa interaction. Raw data from *P. fluorescens* SS101, *massA* mutant, *P. fluorescens* SBW25, *viscA* mutant, *N. americana* alone, and *N. americana* interacting with each of the bacterial strains/mutants were imported individually. The individual datasets were then grouped to allow for the comparisons. In total, the complete data set was comprised of 4126 spectra each with 190454 datapoints in the mass range of 0–5 kDa. The data was processed using the Preprocessing Pipeline of SCiLS Lab 2014b using the default settings. This includes baseline reduction using iterative convolution with 20 interactions and sigma set to 20 and normalization to the total ion count (TIC). Automatic spatial segmentation was used as a first step in data mining. In this approach, similarities between spectra were determined and similar spectra were grouped into a cluster^9^,^57^,^58^. All spectra within a particular cluster were assigned a selected colour and displayed as a spatial segmentation map in which pixels were colour-coded according to their cluster assignment. For each cluster, its spatial region was considered and co-localized ion images were found as correlated to the region with the Pearson correlation of 0.5 or higher; the m/z-values of co-localized ions are listed in Table S2. Individual *m/z* images were created from the selected ions with a hotspot removal applied for better visualization. In order to compare the intensity of ions of interest in different samples, single *m/z* values were also displayed in intensity box plot. The low and high quantiles for the hotspot removal and the intensity box plot were set to 0.00% and 99.00%, correspondingly.

## Additional Information

**How to cite this article**: Song, C. *et al.* Molecular and chemical dialogues in bacteria-protozoa interactions. *Sci. Rep.* **5**, 12837; doi: 10.1038/srep12837 (2015).

## Supplementary Material

Supplementary Information

## Figures and Tables

**Table 1:** Average number of trophozoites (4 replicates) yielded after transferring putrescine-treated cysts to water agar plates with PAS and heat-killed *E. coli*.

Putrescine concentration (mM)	Cysts/μl	Trophozoites/μl
0 (Control)	72.13	79.93
50	18.33	54.17
100	10.00	5.00
150	15.55	4.49
200	25.98	2.64
250	15.41	0.61
300	18.32	0.31
350	14.44	0.09
400	12.54	0.09
450	11.18	0.02
500	14.52	0.01
