# Supplementary material for: Molecular and chemical dialogues in bacteria-protozoa interactions
Source: Sci Rep. 2015 Aug 6;5:12837. doi: 10.1038/srep12837 (PMC4542665; doi:10.1038/srep12837)
Supplement: Supplementary Information [file srep12837-s1.pdf]

Molecular and chemical dialogues in bacteria-protozoa interactions

Chunxu Song[1,8#], Mark Mazzola[2#], Xu Cheng[1], Janina Oetjen[4], Theodore Alexandrov[3,4,5,6,7], Pieter Dorrestein[6], Jeramie Watrous[6], Menno van der Voort[1], Jos M. Raaijmakers[1,8*]

[1]Laboratory of Phytopathology, Wageningen University, 6708 PB Wageningen, the Netherlands

[2]USDA-ARS, 1104 N. Western Ave., Wenatchee, Washington 98801

[3]MALDI Imaging Lab, University of Bremen, 28359 Bremen, Germany

[4]Center for Industrial Mathematics, University of Bremen, 28359 Bremen, Germany

[5]SCiLS GmbH, 28359 Bremen, Germany

[6]Skaggs School of Pharmacy and Pharmaceutical Sciences, University of California at San Diego , San Diego, California 92093, United States

[7]Structural and Computational Biology Unit, European Molecular Biology Laboratory (EMBL), 69117 Heidelberg, Germany

[8]Microbial Ecology Department, Netherlands Institute of Ecology (NIOO-KNAW), 6708PB Wageningen, the Netherlands

[#]These authors contributed equally to this work.

[*]Corresponding author: *j.raaijmakers@nioo.knaw.nl; P:+31 317473497; F:+31 317473675*

Figure S1. The number of genes that are up-regulated (Blue) or down-regulated (Red) in *P. fluorescens* SS101 cells exposed to *N. americana.* The genes are categorized into COGs A thru X (for specification of each of the COGs, see Table S1). Some genes can be placed in more than one COG and thus counted more than once.

Figure S2. MALDI imaging mass spectrometry (IMS) shows no production of massetolide A during the *ΔmassA* mutant-*N. americana* interaction. a.u. = arbitrary units.

Figure S3. Whole genome transcriptome analysis of *P. fluorescens* strains SS101 and SBW25 in the presence of *N. americana*. Heat maps showing log_2-fold changes in the expression of genes that are differentially regulated in both SS101 (A) and SBW25 (B) upon protozoan grazing. Wild type SS101 and SBW25 were grown on 1/5 NBY plates at 25ºC in the presence of *N. americana* for 2-3 days. Cells were collected and total RNA was extracted followed by cDNA synthesis, labelling and hybridization to a SS101/SBW25 whole-genome tiling microarray. The fold changes shown here represent averages of four biological replicates. For a list of all genes differentially regulated in SS101 and SBW25, we refer to Supplementary Tables S1 and S3.

Figure S4. (A) MALDI imaging mass spectrometry (IMS) shows production of viscosin and its derivatives during the *P. fluorescens* SBW25-*N. americana* interaction. a.u. = arbitrary units. (B) MS/MS network analysis and annotation of ion clusters from the *P. fluorescens* SBW25-*N. americana* interaction. Ion clusters in the black square represents the lipopeptide viscosin and its derivatives; the black circle represents the 325 m/z ion cluster; the grey square represents the 766 m/z ion cluster. (C) MS/MS analysis further indicated that the parent ion

with 1148.70 m/z detected in the *P. fluorescens* SBW25- *N. americana* interaction is most likely viscosin.

Figure S5. Box plots depicting the production of viscosin and its derivatives in *P. fluorescens* SBW25 alone, *N. americana* alone, *P. fluorescens* SBW25-*N. americana* interaction and *viscA* mutant alone. The box plots represent the median intensity in arbitrary units after TIC normalization (horizontal line), the upper and lower quartiles (box layout, spectra in which the intensities are within a range of 25% - 75% of the data), the upper and lower quantiles (dashed lines, spectra in which the intensities are within a range of 1% - 99%) as well as the outliers (spectra with intensities greater than 99% and lower than 1% of the data).

Figure S6. MALDI imaging mass spectrometry (IMS) shows production of 88 m/z ions in the *P. fluorescens* SS101-*N. americana* (A) and *P. fluorescens* SBW25-*N. americana* (B) interactions respectively. a.u. = arbitrary units.

Figure S7. (A) MALDI imaging mass spectrometry (IMS) shows production of 311-477 m/z ions and its cluster ions in the MS/MS network in *P. fluorescens* SBW25-*N. americana* interaction. a.u. = arbitrary units. (B) MS/MS profile of 325 m/z during the *P. fluorescens* SBW25-*N. americana.* (C) MALDI imaging mass spectrometry (IMS) shows production of 752-809 m/z ions and its cluster ions in the MS/MS network in *P. fluorescens* SBW25-*N. americana* interaction. a.u. = arbitrary units. (D) MS/MS profile of 766 m/z during the *P. fluorescens* SBW25-*N. americana*

Figure S8. MS/MS profile of 843 m/z (A) and 868 m/z (B) during the *P. fluorescens* SBW25-*N. americana* interaction.

Figure S9. MS/MS profile of 883 m/z (A) and 897 m/z (B) during the *P. fluorescens* SBW25-*N. americana* interaction.

Table S1. Whole genome transcriptome analysis of *P. fluorescens* strain SS101 in the presence of *N. americana*

Table S2. Co-localization ions of the MALDI-IMS of *P. fluorescens* strain SS101 in the presence of *N. americana*

Table S3. Whole genome transcriptome analysis of *P. fluorescens* strain SBW25 in the presence of *N. americana*

Table S4. Ion clusters of the lipopeptide massetolide A and its derivatives in *P. fluorescens* strain SS101 interaction with *N. americana*

Table S5. Ion cluster of 325-477 m/z in *P. fluorescens* strain SS101 interaction with *N. americana*

Table S6. Ion cluster of 766-796 m/z in *P. fluorescens* strain SS101 interaction with *N. americana*

Figure S1

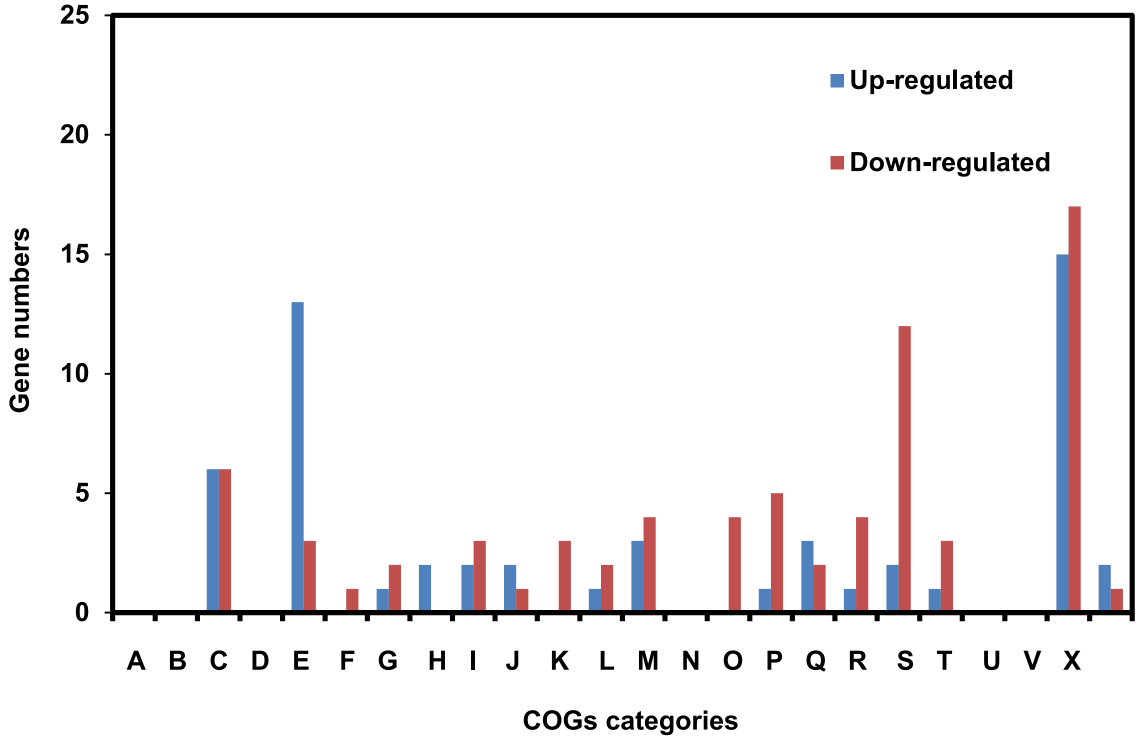
Up-regulated
Down-regulated
Gene numbers
0
5
10
15
20
25
A B C D E F G H I J K L M N O P Q R S T U V X
COGs categories

Figure S2

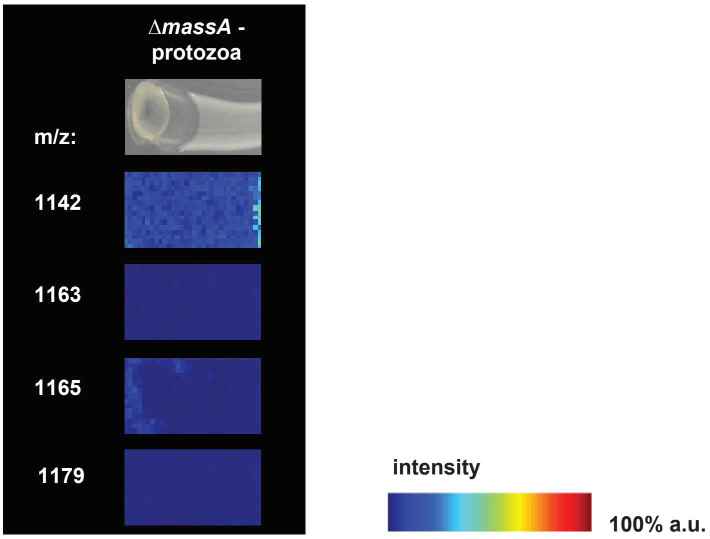

Figure S3

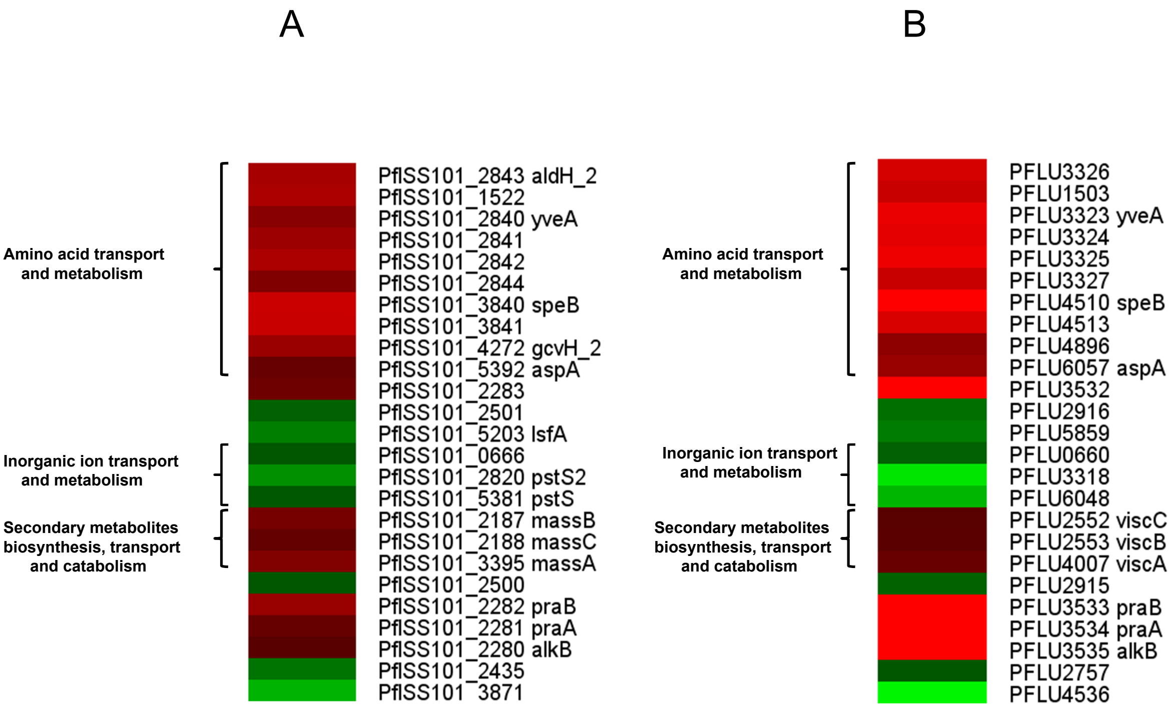
A
B
Amino acid transport and metabolism
Inorganic ion transport and metabolism
Secondary metabolites biosynthesis, transport and catabolism
PflSS101_2843 aldH_2
PflSS101_1522
PflSS101_2840 yveA
PflSS101_2841
PflSS101_2842
PflSS101_2844
PflSS101_3840 speB
PflSS101_3841
PflSS101_4272 gcvH_2
PflSS101_5392 aspA
PflSS101_2283
PflSS101_2501
PflSS101_5203 lsfA
PflSS101_0666
PflSS101_2820 pstS2
PflSS101_5381 pstS
PflSS101_2187 massB
PflSS101_2188 massC
PflSS101_3395 massA
PflSS101_2500
PflSS101_2282 praB
PflSS101_2281 praA
PflSS101_2280 alkB
PflSS101_2435
PflSS101_3871
PFLU3326
PFLU1503
PFLU3323 yveA
PFLU3324
PFLU3325
PFLU3327
PFLU4510 speB
PFLU4513
PFLU4896
PFLU6057 aspA
PFLU3532
PFLU2916
PFLU5859
PFLU0660
PFLU3318
PFLU6048
PFLU2552 viscC
PFLU2553 viscB
PFLU4007 viscA
PFLU2915
PFLU3533 praB
PFLU3534 praA
PFLU3535 alkB
PFLU2757
PFLU4536

Figure S4

A

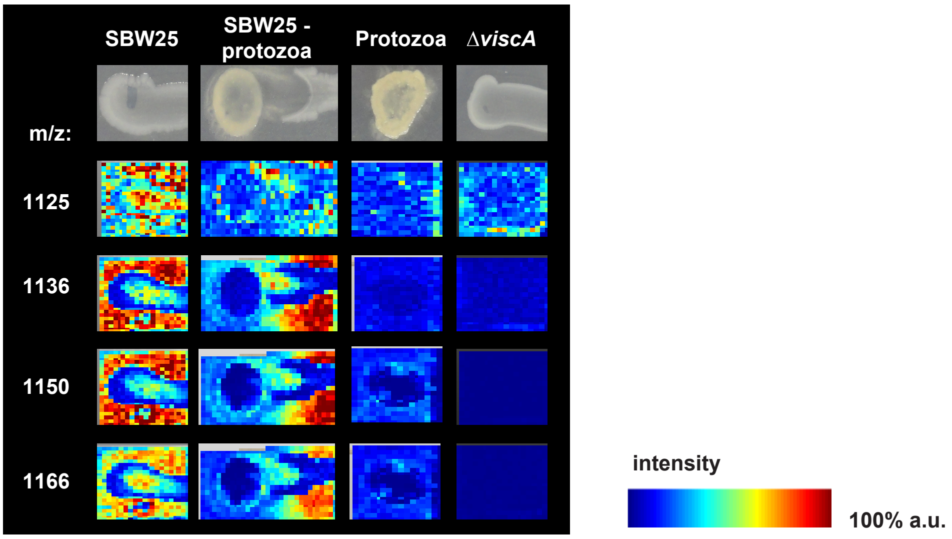

B

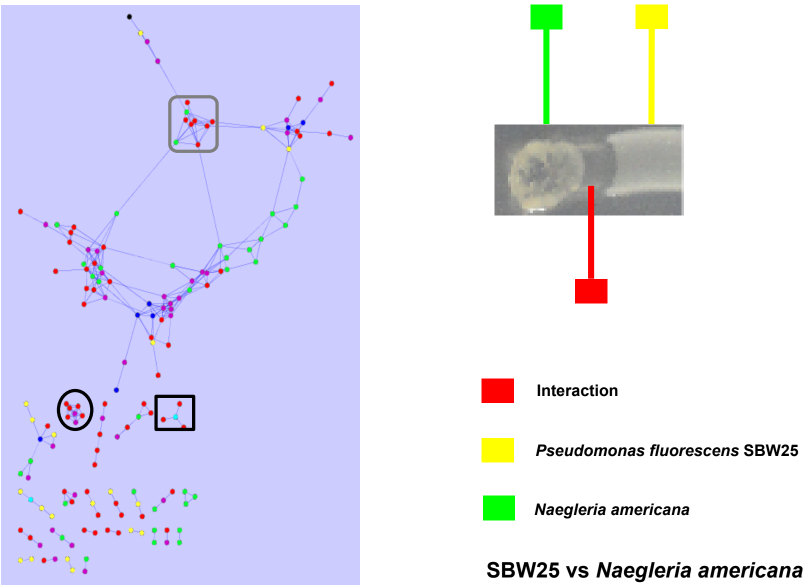

C

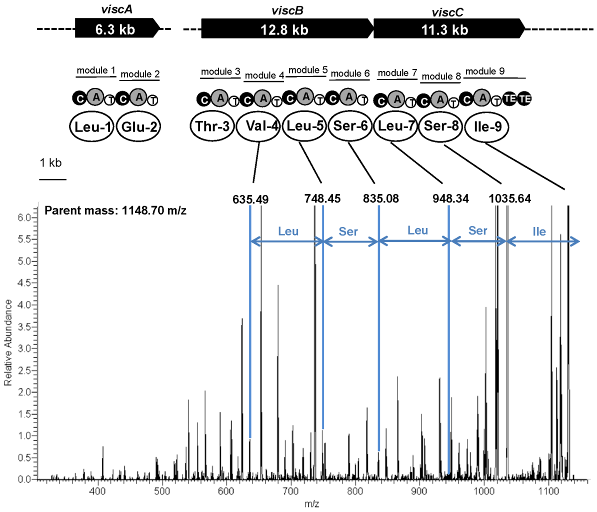

Figure S5

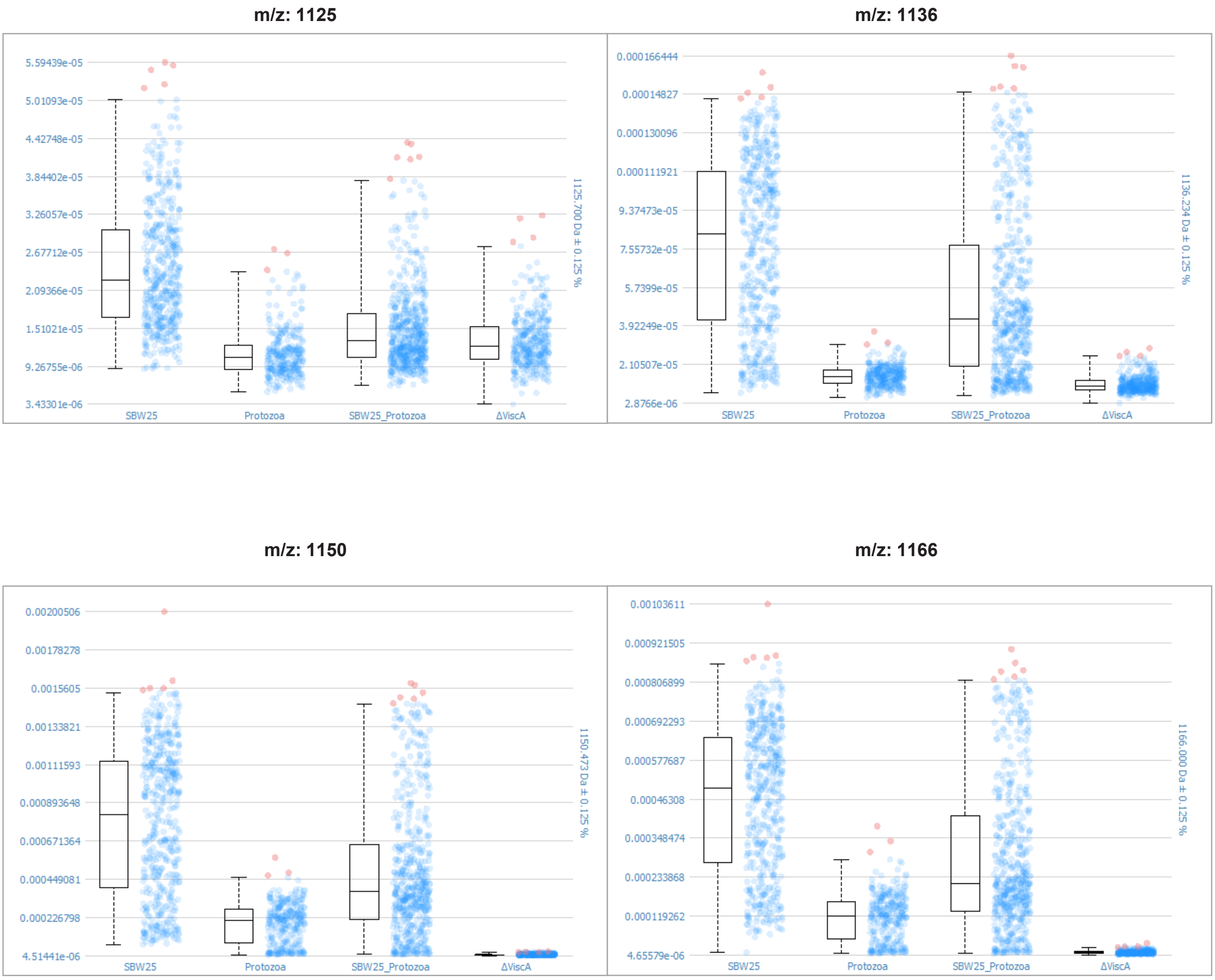
m/z: 1125
5.59439e-05
5.01093e-05
4.42748e-05
3.84402e-05
3.26057e-05
2.67712e-05
2.09366e-05
1.51021e-05
9.26755e-06
3.43301e-06
1125.700 Da ± 0.125 %
SBW25
Protozoa
SBW25_Protozoa
ΔViscA
m/z: 1136
0.000166444
0.00014827
0.000130096
0.000111921
9.37473e-05
7.55732e-05
5.7399e-05
3.92249e-05
2.10507e-05
2.8766e-06
1136.234 Da ± 0.125 %
SBW25
Protozoa
SBW25_Protozoa
ΔViscA
m/z: 1150
0.00200506
0.00178278
0.0015605
0.00133821
0.00111593
0.000893648
0.000671364
0.000449081
0.000226798
4.51441e-06
1150.473 Da ± 0.125 %
SBW25
Protozoa
SBW25_Protozoa
ΔViscA
m/z: 1166
0.00103611
0.000921505
0.000806899
0.000692293
0.000577687
0.00046308
0.000348474
0.000233868
0.000119262
4.65579e-06
1166.000 Da ± 0.125 %
SBW25
Protozoa
SBW25_Protozoa
ΔViscA

Figure S6

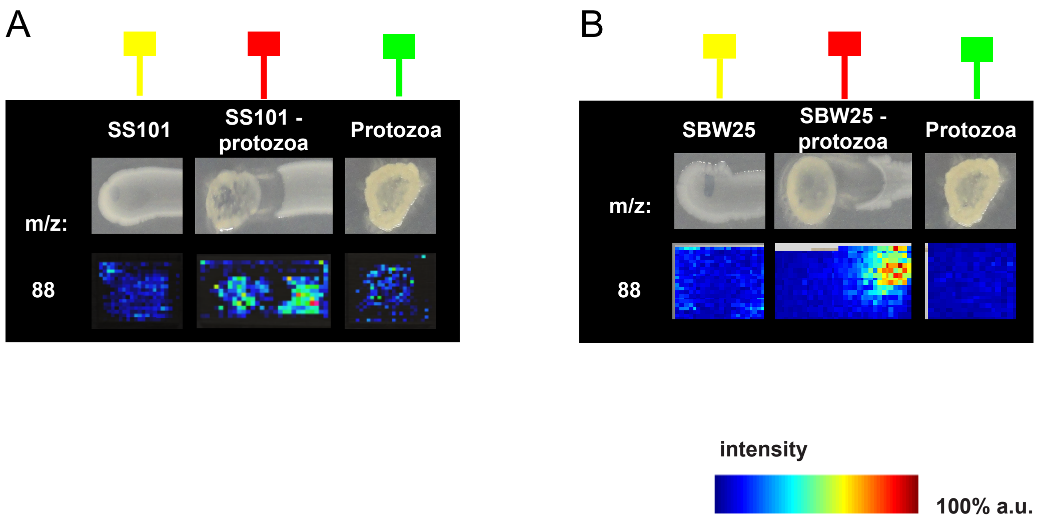
A
SS101
SS101 - protozoa
Protozoa
m/z:
88
B
SBW25
SBW25 - protozoa
Protozoa
m/z:
88
intensity
100% a.u.

Figure S7

A

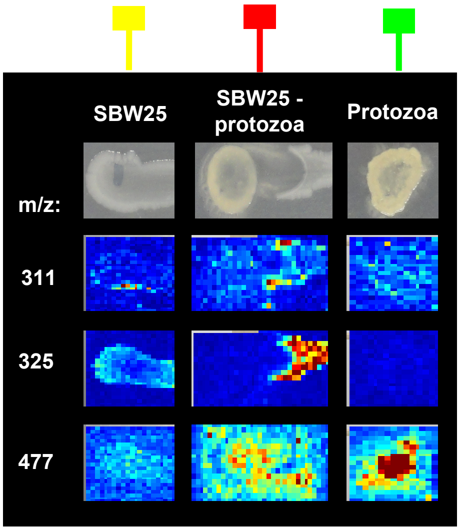

B

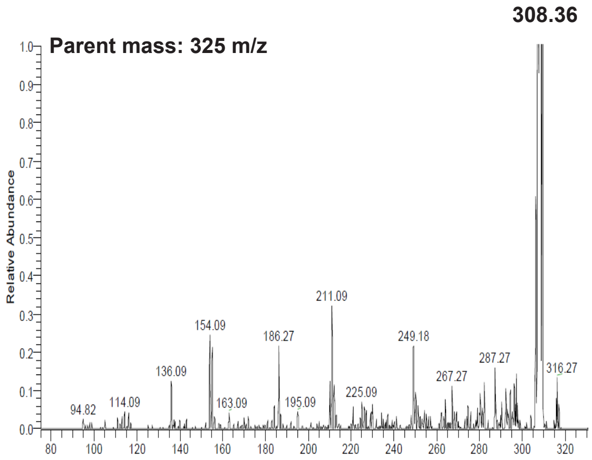

C

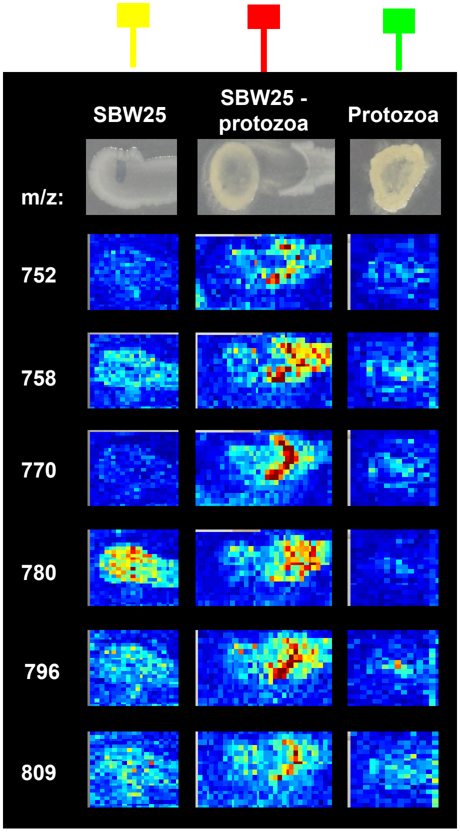

D

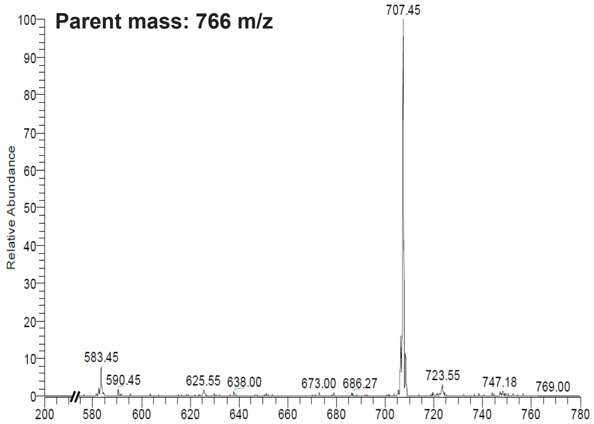

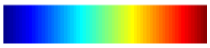

Figure S8

A

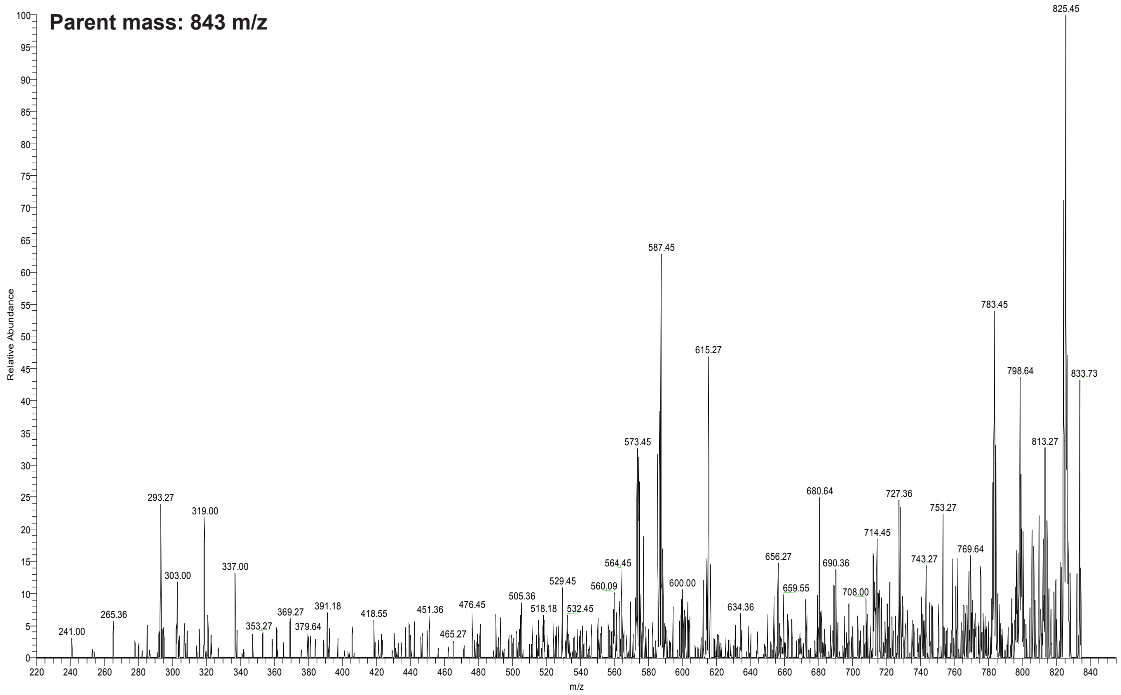
Parent mass: 843 m/z
Relative Abundance
m/z
825.45
587.45
783.45
615.27
798.64
833.73
573.45
813.27
293.27
680.64
727.36
319.00
753.27
714.45
337.00
656.27
743.27
769.64
564.45
690.36
303.00
529.45
560.09
600.00
659.55
708.00
505.36
476.45
391.18
451.36
518.18
532.45
634.36
369.27
418.55
265.36
353.27
379.64
241.00
465.27

B

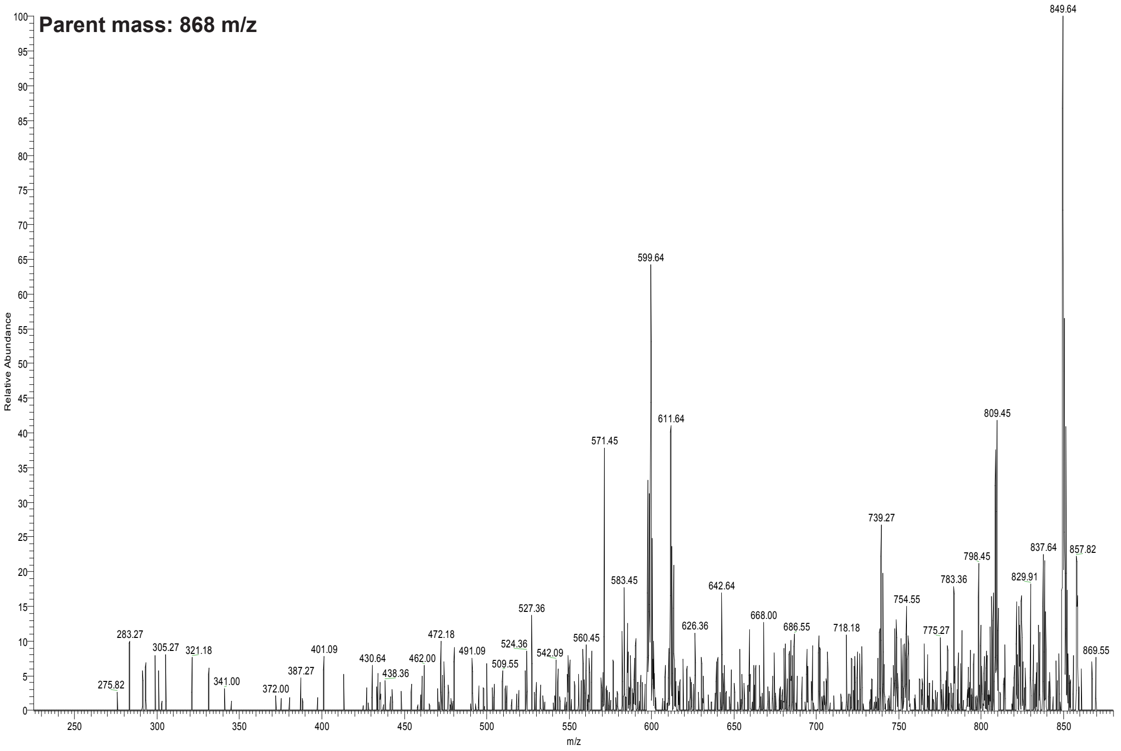
Parent mass: 868 m/z
Relative Abundance
m/z
849.64
599.64
809.45
611.64
571.45
739.27
837.64
857.82
798.45
829.91
583.45
783.36
642.64
754.55
527.36
668.00
686.55
718.18
775.27
560.45
626.36
283.27
472.18
305.27
321.18
401.09
524.36
491.09
542.09
462.00
430.64
509.55
869.55
387.27
438.36
341.00
275.82
372.00

Figure S9

A

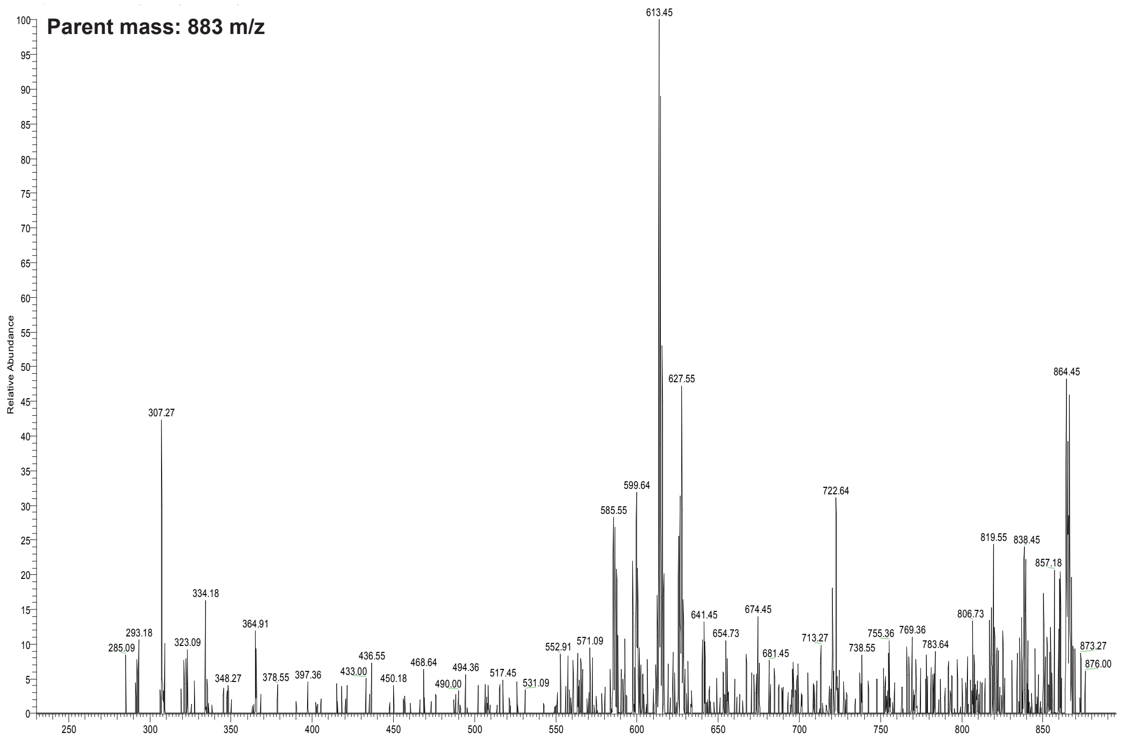

Parent mass: 883 m/z
Relative Abundance
m/z
613.45
627.55
864.45
307.27
599.64
585.55
722.64
819.55
838.45
857.18
334.18
364.91
293.18
285.09
323.09
641.45
674.45
654.73
806.73
713.27
755.36
769.36
783.64
738.55
681.45
873.27
876.00
348.27
378.55
397.36
433.00
436.55
450.18
468.64
490.00
494.36
517.45
531.09
552.91
571.09

B

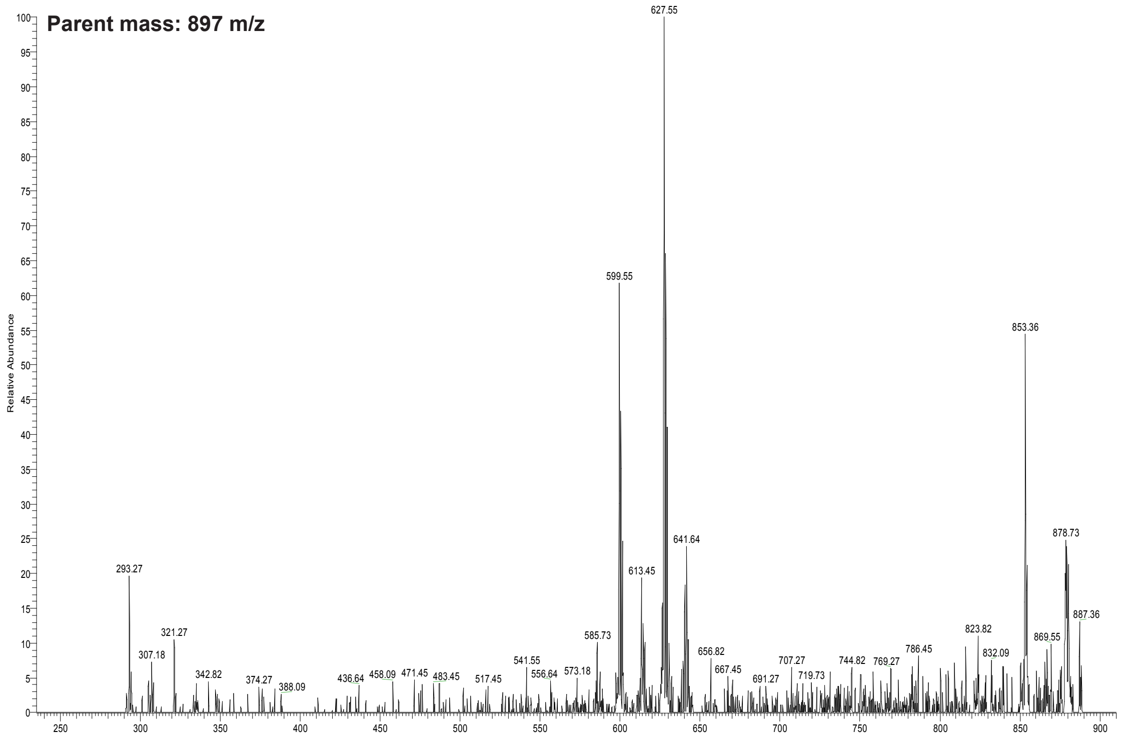

Parent mass: 897 m/z
Relative Abundance
m/z
627.55
599.55
853.36
878.73
641.64
613.45
293.27
321.27
307.18
887.36
823.82
832.09
869.55
786.45
585.73
656.82
667.45
707.27
744.82
769.27
719.73
691.27
541.55
556.64
573.18
342.82
374.27
388.09
436.64
458.09
471.45
483.45
517.45

Locustag	Fold change		P value-FDR	Gene names	Gene description	COG	COG description
PflSS101_0103	2.385765679	down	0.0299		conserved hypothetical protein	X	Not in COGs
PflSS101_0137	2.047757654	down	0.0141		RNA polymerase sigma-70 factor, ECF family	K	Transcription
PflSS101_0176	2.095470647	down	0.00342		von Willebrand factor type A domain protein	R	General function prediction only
PflSS101_0230	2.28208116	down	0.0252		histidine triad domain protein	F	Nucleotide transport and metabolism
PflSS101_0314	2.193433447	down	0.0392		transporter, major facilitator family	G	Carbohydrate transport and metabolism
PflSS101_0475	2.486267567	up	0.0196	putP	sodium_proline symporter	E	Amino acid transport and metabolism
PflSS101_0551	2.332362652	up	0.0103	rplI	ribosomal protein L9	J	Translation
PflSS101_0566	3.097485781	up	0.00123	pcd	piperideine-6-carboxylate dehydrogenase	C	Energy production and conversion
PflSS101_0567	2.722925425	up	0.00383		FAD dependent oxidoreductase	E	Amino acid transport and metabolism
PflSS101_0606	2.050752163	up	0.00753	oprC	TonB-dependent outermembrane copper receptor OprC	P	Inorganic ion transport and metabolism
PflSS101_0666	2.043928112	down	0.0483		putative copper chaperone CopZ	P	Inorganic ion transport and metabolism
PflSS101_0697	2.463279917	down	0.0177		conserved hypothetical protein	X	Not in COGs
PflSS101_0855	2.434810978	down	0.00247		PF04402 family protein	S	Function unknown
PflSS101_0875	3.788087919	down	0.00182	kdgD	5-dehydro-4-deoxyglucarate dehydratase	E	Amino acid transport and metabolism
PflSS101_0876	3.51623279	down	0.000928		aldehyde dehydrogenase family protein	C	Energy production and conversion
PflSS101_1118	2.140944484	down	0.0443	rpmE_2	ribosomal protein L31	J	Translation
PflSS101_1337	2.221079343	down	0.00851	dctA	aerobic C4-dicarboxylate transport protein	C	Energy production and conversion
PflSS101_1413	2.047787905	up	0.012	adhB	alcohol dehydrogenase II	C	Energy production and conversion
PflSS101_1522	4.090725899	up	0.00225		putative thiamine pyrophosphate enzyme	E	Amino acid transport and metabolism
PflSS101_1544	2.008988422	down	0.0423		conserved hypothetical protein	X	Not in COGs
PflSS101_1559	2.504130112	down	0.0151		conserved hypothetical protein	M	Cell wall/membrane biogenesis
PflSS101_1574	2.030911904	down	0.00915	fadA	acetyl-CoA C-acyltransferase FadA	I	Lipid transport and metabolism
PflSS101_1618	9.950917244	up	0.00185		conserved hypothetical protein	X	Not in COGs
PflSS101_1619	6.251049519	up	0.00241		conserved hypothetical protein	X	Not in COGs
PflSS101_1620	4.24153614	up	0.00232		integral membrane protein, PF00892 family	X	Not in COGs
PflSS101_1621	3.246046782	up	0.00679		putative lipoprotein	X	Not in COGs
PflSS101_1709	2.046893358	up	0.0223	oprI	outer membrane lipoprotein OprI	X	Not in COGs
PflSS101_1727	2.330752386	down	0.0361		conserved domain protein	S	Function unknown
PflSS101_1998	2.754859345	down	0.00518		thioesterase family protein	Q	Secondary metabolites biosynthesis, transport and catabolism
PflSS101_2013	2.290246395	down	0.00453		conserved hypothetical protein	S	Function unknown
PflSS101_2057	2.653518132	down	0.0174		conserved hypothetical protein	X	Not in COGs
PflSS101_2063	2.082899888	down	0.00829		oxidoreductase, short chain dehydrogenase/reductase family	R	General function prediction only
PflSS101_2065	2.468221669	down	0.0208		putative membrane protein, PF07300 family	S	Function unknown
PflSS101_2089	2.038626598	down	0.00885		conserved hypothetical protein	X	Not in COGs
PflSS101_2090	2.562253545	down	0.0225		conserved hypothetical protein	X	Not in COGs
PflSS101_2092	2.377497433	down	0.00286		ATP-dependent Clp protease, proteolytic subunit ClpP-like protein	O	Posttranslational modification, protein turnover, chaperones
PflSS101_2094	2.132504391	down	0.00674		conserved domain protein	X	Not in COGs
PflSS101_2098	2.373336585	down	0.0223		hypothetical protein	X	Not in COGs
PflSS101_2099	2.664422548	down	0.00912		conserved hypothetical protein	S	Function unknown
PflSS101_2187	2.641467571	up	0.00353	massB	non-ribosomal peptide synthetase MassB	Q	Secondary metabolites biosynthesis, transport and catabolism
PflSS101_2188	2.275959492	up	0.00388	massC	non-ribosomal peptide synthetase MassC	Q	Secondary metabolites biosynthesis, transport and catabolism
PflSS101_2192	4.550174713	up	0.00235	megL	methionine gamma-lyase	E	Amino acid transport and metabolism
PflSS101_2195	2.409641981	up	0.00902	alkG	rubredoxin 2	C	Energy production and conversion
PflSS101_2196	2.501330853	up	0.00899		AP endonuclease, family 2	G	Carbohydrate transport and metabolism
PflSS101_2210	2.359759951	down	0.00931	budC	diacetyl reductase S-acetoin forming	I	Lipid transport and metabolism
PflSS101_2211	2.033169922	down	0.0035	acoR	acetoin catabolism regulatory protein	Q	Secondary metabolites biosynthesis, transport and catabolism
PflSS101_2212	8.304666507	down	0.00385	acoA	acetoin dehydrogenase E1 component, alpha subunit	C	Energy production and conversion
PflSS101_2213	7.958622527	down	0.00597	acoB	acetoin dehydrogenase E1 component, beta subunit	C	Energy production and conversion
PflSS101_2214	6.834233378	down	0.00561	acoC	acetoin dehydrogenase E2 component, dihydrolipoamide acetyltransferase	R	General function prediction only
PflSS101_2280	2.072992563	up	0.0262	alkB	alkane 1-monooxygenase	X	Not in COGs
PflSS101_2281	2.323854923	up	0.0119	praA	protein activator of alkane oxidation PraA	X	Not in COGs
PflSS101_2282	3.498092413	up	0.00551	praB	protein activator of alkane oxidation PraB	X	Not in COGs
PflSS101_2283	2.478863239	up	0.0189		putative outer membrane protein	I	Lipid transport and metabolism
PflSS101_2291	2.107859135	up	0.00414		BNR_Asp-box repeat domain protein	R	General function prediction only
PflSS101_2340	2.64029376	down	0.00198		conserved hypothetical protein	X	Not in COGs
PflSS101_2379	2.233312157	down	0.0332		conserved hypothetical protein	S	Function unknown
PflSS101_2435	2.576583889	down	0.0158		conserved hypothetical protein	X	Not in COGs
PflSS101_2436	2.446096538	down	0.0152		Mg2+ transporter-C, MgtC family	S	Function unknown
PflSS101_2496	2.138371789	down	0.00694	ligD	DNA ligase D	L	Replication, recombination and repair
PflSS101_2497	2.264539086	down	0.0096		Ku protein	S	Function unknown
PflSS101_2500	2.038978788	down	0.00243		conserved hypothetical protein	S	Function unknown
PflSS101_2501	2.234040604	down	0.00078		transcriptional regulator, TetR family	K	Transcription
PflSS101_2560	2.469946384	up	0.00233	aprA	extracellular alkaline metalloprotease AprA	X	Not in COGs
PflSS101_2575	2.571957152	down	0.0256		conserved hypothetical protein	X	Not in COGs
PflSS101_2577	2.32819962	down	0.0188		transporter, CPA2 family	P	Inorganic ion transport and metabolism
PflSS101_2591	2.039049786	down	0.00886		transcriptional regulator, LuxR family	T	Signal transduction mechanisms
PflSS101_2598	2.762475967	up	0.00914		formyl transferase domain_enoyl-CoA hydratase/isomerase family protein	J	Translation
PflSS101_2627	2.473874361	down	0.00634		putative KaiC domain protein	T	Signal transduction mechanisms
PflSS101_2683	2.146178222	down	0.0142		putative DNA topoisomerase I	L	Replication, recombination and repair
PflSS101_2820	3.261658451	down	0.0141	pstS2	phosphate ABC transporter, phosphate-binding protein PstS2	P	Inorganic ion transport and metabolism
PflSS101_2840	3.091795206	up	0.000763	yveA	aspartate_proton symporter YveA	E	Amino acid transport and metabolism
PflSS101_2841	3.630767107	up	0.00124		proline racemase family protein	E	Amino acid transport and metabolism
PflSS101_2842	4.097471237	up	0.000389		dihydrodipicolinate synthetase family protein	E	Amino acid transport and metabolism
PflSS101_2843	3.92638731	up	0.00188	aldH_2	NADP-dependent fatty aldehyde dehydrogenase	C	Energy production and conversion
PflSS101_2844	2.884896994	up	0.00236		FAD dependent oxidoreductase	E	Amino acid transport and metabolism
PflSS101_2914	2.191737652	up	0.00388		conserved hypothetical protein	X	Not in COGs
PflSS101_2982	2.611388206	up	0.00658		putative lipoprotein	X	Not in COGs
PflSS101_3033	2.116470081	down	0.00635		amino acid ABC transporter, permease protein, 3-TM region, His/Glu/Gln/Arg	E	Amino acid transport and metabolism
PflSS101_3049	2.472496271	up	0.00305		conserved hypothetical protein	S	Function unknown
PflSS101_3152	2.160084301	down	0.0245		tRNA-Ser	0	0
PflSS101_3183	2.443406706	down	0.00722		glutathione S-transferase, C-terminal domain protein	O	Posttranslational modification, protein turnover, chaperones
PflSS101_3223	2.341401274	down	0.0392		conserved hypothetical protein	X	Not in COGs
PflSS101_3224	2.714154895	down	0.00567		conserved hypothetical protein	X	Not in COGs
PflSS101_3225	2.485192984	down	0.0121		hypothetical protein	X	Not in COGs
PflSS101_3238	2.01956863	down	0.0124		conserved hypothetical protein	S	Function unknown
PflSS101_3334	2.604944798	down	0.00307		NAD dependent epimerase_dehydratase family protein	M	Cell wall/membrane biogenesis
PflSS101_3335	2.356566029	down	0.00245		SMP-30_Gluconolaconase_LRE family protein	G	Carbohydrate transport and metabolism
PflSS101_3395	2.887057781	up	0.00085	massA	non-ribosomal peptide synthetase MassA	Q	Secondary metabolites biosynthesis, transport and catabolism
PflSS101_3396	2.464760303	up	0.000475		transcriptional regulator, LuxR family	T	Signal transduction mechanisms
PflSS101_3398	2.182132244	up	0.000494	nodT	efflux transporter, outer membrane factor lipoprotein, NodT family	M	Cell wall/membrane biogenesis
PflSS101_3564	2.377555609	up	0.0223		conserved hypothetical protein	X	Not in COGs
PflSS101_3655	2.143716195	down	0.0415		conserved hypothetical protein	X	Not in COGs
PflSS101_3821	2.063116312	up	0.00383		NlpC_P60 family protein	M	Cell wall/membrane biogenesis
PflSS101_3840	5.277371883	up	0.00166	speB	agmatinase	E	Amino acid transport and metabolism
PflSS101_3841	5.169645309	up	0.00129		transporter, SSS family	E	Amino acid transport and metabolism
PflSS101_3854	2.9566411	down	0.0254		conserved hypothetical protein	X	Not in COGs
PflSS101_3871	4.32712371	down	0.00548		conserved hypothetical protein	X	Not in COGs
PflSS101_3883	3.583265305	up	0.0157	ccoO_1	cytochrome c oxidase, cbb3-type, subunit II	C	Energy production and conversion
PflSS101_3897	2.74943924	up	0.00602	hemN	coproporphyrinogen dehydrogenase	H	Coenzyme transport and metabolism
PflSS101_3938	2.07582736	up	0.0337		PF04320 family protein	S	Function unknown
PflSS101_4081	2.476959467	up	0.0159		PhrS RNA	0	0
PflSS101_4103	3.1138134	up	0.00676		short chain dehydrogenase_reductase family protein	I	Lipid transport and metabolism
PflSS101_4143	2.091638565	up	0.00385	ribH_2	6,7-dimethyl-8-ribityllumazine synthase	H	Coenzyme transport and metabolism
PflSS101_4163	2.098148883	down	0.00636		HAD hydrolase, family IB	E	Amino acid transport and metabolism
PflSS101_4250	2.047661304	up	0.00127		conserved hypothetical protein	X	Not in COGs
PflSS101_4272	3.595456362	up	0.00267	gcvH_2	glycine cleavage system H protein	E	Amino acid transport and metabolism
PflSS101_4273	2.157778502	up	0.00248	gcvP	glycine dehydrogenase	E	Amino acid transport and metabolism
PflSS101_4366	2.242273808	up	0.0374		conserved hypothetical protein	X	Not in COGs
PflSS101_4477	2.242925474	down	0.0133		response regulator	T	Signal transduction mechanisms
PflSS101_4540	2.158946861	down	0.0212		NAD dependent epimerase_dehydratase family protein	M	Cell wall/membrane biogenesis
PflSS101_4671	2.322312377	down	0.00921		elongation factor, GreA_GreB family	K	Transcription
PflSS101_4697	2.192515603	down	0.01		aldehyde oxidase and xanthine dehydrogenase family protein	C	Energy production and conversion
PflSS101_4699	2.097925741	down	0.0125		2Fe-2S iron-sulfur cluster binding domain protein	C	Energy production and conversion
PflSS101_4738	2.161044359	up	0.018		sRNA P24	0	0
PflSS101_4796	2.671133757	up	0.00506	ppa	inorganic diphosphatase	C	Energy production and conversion
PflSS101_4811	2.225212336	up	0.0192	xseB	exodeoxyribonuclease VII, small subunit	L	Replication, recombination and repair
PflSS101_4834	2.567535639	up	0.00376	oprG	outer membrane protein OprG	M	Cell wall/membrane biogenesis
PflSS101_4837	2.639831168	down	0.00822		PF01904 family protein	S	Function unknown
PflSS101_4838	2.435756391	down	0.00852		endonuclease_exonuclease_phosphatase family protein	R	General function prediction only
PflSS101_4839	2.374706173	down	0.00634		putative cardiolipin synthetase YbhO	I	Lipid transport and metabolism
PflSS101_4840	2.792438227	down	0.00884		putative membrane protein	S	Function unknown
PflSS101_4927	2.054293657	down	0.0103		DnaJ domain protein	O	Posttranslational modification, protein turnover, chaperones
PflSS101_5159	2.145127783	down	0.0415	cadA_1	cadmium-exporting ATPase	P	Inorganic ion transport and metabolism
PflSS101_5203	2.816554857	down	0.0207	lsfA	thiol-specific antioxidant protein LsfA	O	Posttranslational modification, protein turnover, chaperones
PflSS101_5210	4.734334946	up	0.00293		conserved hypothetical protein	X	Not in COGs
PflSS101_5381	2.081254081	down	0.00566	pstS	phosphate ABC transporter, periplasmic phosphate-binding protein	P	Inorganic ion transport and metabolism
PflSS101_5392	2.344167709	up	0.00171	aspA	aspartate ammonia-lyase	E	Amino acid transport and metabolism
PflSS101_5454	2.056929389	down	0.0218		cyclopropane-fatty-acyl-phospholipid synthase	M	Cell wall/membrane biogenesis

Light blue class

m/z at peak centroid	± m/z interval	m/z with maximal correlation value	Maximal correlation value
994.215	1.243	994.18	0.507
1136.038	1.42	1136.063	0.672
1142.68	1.428	1142.667	0.579
1150.44	1.779	1151.032	0.727
1165.377	1.457	1165.796	0.606
1181.805	1.477	1181.856	0.539

Green class

m/z at peak centroid	± m/z interval	m/z with maximal correlation value	Maximal correlation value
249.575	0.312	249.575	0.559
265.578	0.332	265.548	0.588
445.112	0.556	445.12	0.543
461.112	0.576	461.136	0.595
476.833	0.335	477.16	0.518
477.503	0.335	477.225	0.54
672.38	0.84	672.361	0.588
688.539	0.861	688.519	0.511
740.469	0.473	740.922	0.517
741.415	0.473	740.962	0.504
766.87	0.959	766.87	0.549
1163.397	1.454	1163.003	0.676
1179.645	1.475	1179.121	0.699
1201.933	1.502	1201.43	0.661

Orange class

m/z at peak centroid	± m/z interval	m/z with maximal correlation value	Maximal correlation value
183.895	0.23	183.95	0.512
223.928	0.28	223.917	0.568
257.939	0.173	258.089	0.502
258.286	0.173	258.136	0.504

Dark red class

m/z at peak centroid	± m/z interval	m/z with maximal correlation value	Maximal correlation value
43.338	0.229	43.25	0.687
43.611	0.045	43.567	0.549
88.292	0.064	88.339	0.519
88.42	0.064	88.374	0.502
194.579	0.243	194.579	0.568
232.825	0.291	232.774	0.76
281.931	0.352	281.956	0.529
284.791	0.195	284.951	0.506
285.181	0.195	285.039	0.526
313.524	0.768	313.136	0.857
325.504	0.676	325.27	0.828
326.495	0.314	326.207	0.585
328.747	0.221	328.944	0.523
329	0.032	329.012	0.513
329.254	0.223	329.039	0.525
353.334	0.442	353.251	0.623
369.37	0.462	369.249	0.776
373.272	0.467	373.179	0.539
454.097	0.292	454.373	0.512
454.68	0.292	454.404	0.53
552.543	1.152	551.949	0.871
555.688	0.695	555.705	0.516
564.888	1.562	564.12	0.896
576.032	0.72	575.96	0.753
592.353	0.876	592.217	0.847
604.505	1.013	604.13	0.877
616.27	0.491	616.549	0.572
616.955	0.194	617.121	0.561
617.622	0.474	617.213	0.565
626.435	0.836	626.193	0.839
648.201	0.81	648.219	0.77
684.044	0.855	684.063	0.605
703.918	0.46	704.339	0.507
704.58	0.202	704.713	0.637
705.406	0.623	704.812	0.639
715.294	1.162	714.767	0.88
727.495	1.162	727.535	0.885
737.188	0.642	737.811	0.571
738.199	0.368	738.416	0.678
739.216	0.649	738.618	0.682
743.482	0.929	743.675	0.847
747.093	0.934	747.164	0.664
750.631	1.293	750.498	0.769
765.276	0.957	765.307	0.683
769.257	0.507	769.753	0.52
770.271	0.507	769.774	0.514
780.708	0.976	780.677	0.52
790.017	0.988	789.985	0.511
957.25	1.197	957.296	0.569

Locustag	Genes	Gene descriptions	Fold change		P value-FDR
PFLU0062		hypothetical protein	2.141166897	down	0.000867
PFLU0630		hypothetical protein	2.038700049	down	0.00127
PFLU0640		putative two-component system response regulator	2.091669342	down	0.000179
PFLU0660		putative substrate-binding periplasmic protein	2.23616114	down	0.000241
PFLU0675		beta alanine--pyruvate transaminase	3.459464312	up	0.00124
PFLU0676	mmsA	methylmalonate-semialdehyde dehydrogenase	3.210054874	up	0.00123
PFLU0873		hypothetical protein	3.903486906	down	0.000852
PFLU0874	fumC	fumarate hydratase	2.935147152	down	0.0002
PFLU0875		hypothetical protein	2.664475865	down	0.000599
PFLU0876	sodA	superoxide dismutase	2.771515851	down	0.000876
PFLU0907		hypothetical protein	4.870885525	down	0.0108
PFLU0954		hypothetical protein	2.756617184	down	0.000715
PFLU0955		hypothetical protein	2.412969953	down	0.00146
PFLU0990	algD	GDP-mannose 6-dehydrogenase	2.210777521	up	0.000406
PFLU1050		putative DNA-binding protein	2.129960537	up	0.00086
PFLU1172		hypothetical protein	2.032741658	down	0.00426
PFLU1174		putative phage tail-like protein	2.459479616	down	0.019
PFLU1176		putative phage-like protein	2.092829914	down	0.00444
PFLU1177		hypothetical protein	2.530116817	down	0.0228
PFLU1178		putative phage tail-like protein	2.617762453	down	0.0166
PFLU1179		putative phage tail-like protein	2.519406781	down	0.00834
PFLU1180		putative phage hypothetical protein	2.277522532	down	0.015
PFLU1181		putative phage hypothetical protein	2.851199211	down	0.00912
PFLU1366	pcaH	protocatechuate 3,4-dioxygenase subunit beta	2.621680021	up	0.00544
PFLU1367	pcaG	protocatechuate 3,4-dioxygenase alpha chain	2.141105413	up	0.00234
PFLU1391		putative acyltransferase	2.392966307	down	0.00868
PFLU1392		hypothetical protein	3.390410538	down	0.0026
PFLU1439		putative lipoprotein	2.539231007	down	0.000164
PFLU1448		putative transporter-like membrane protein	4.535796642	up	0.00216
PFLU1449		putative transporter-like exported protein	3.014289618	up	0.00483
PFLU1450		hypothetical protein	3.103611231	up	0.00501
PFLU1492		hypothetical protein	2.39845627	down	0.00124
PFLU1493		hypothetical protein	2.494904116	down	0.00079
PFLU1502		putative dehydrogenase	4.06276083	up	0.000163
PFLU1503		hypothetical protein	5.193297386	up	0.0002
PFLU1551		hypothetical protein	2.116040171	down	0.00024
PFLU1771		putative p-aminobenzoic acid synthase	2.249596357	up	0.0117
PFLU1772		putative chorismate mutase/prephenate dehydrogenase	2.313663721	up	0.0156
PFLU1773		hypothetical protein	2.44193697	up	0.00918
PFLU1774		putative methyltransferase	2.454258442	up	0.00444
PFLU1775		putative oxidoreductase	2.613559961	up	0.0064
PFLU1778	phnD	phosphonates ABC transporter substrate-binding periplasmic protein	2.01677822	down	0.0124
PFLU1791		hypothetical protein	2.181700673	down	0.000181
PFLU2021		putative transporter-like membrane protein	4.98244381	up	0.0000529
PFLU2022		putative dipeptidyl peptidase	7.180238724	up	0.0000443
PFLU2023		putative LysR family regulatory protein	5.214242458	up	0.0000955
PFLU2054		hypothetical protein	2.779976638	down	0.000687
PFLU2091		hypothetical protein	2.029107809	up	0.000873
PFLU2092		hypothetical protein	2.070543528	up	0.000846
PFLU2093		putative dehydrogenase	2.17754221	up	0.000675
PFLU2352		putative oxidoreductase	7.034337182	down	0.000875
PFLU2353		hypothetical protein	6.098324909	down	0.00109
PFLU2354		putative aldolase	6.664996566	down	0.000486
PFLU2355		putative transporter-like membrane protein	7.341900743	down	0.000569
PFLU2356		putative isomerase	4.724287904	down	0.0008
PFLU2471	tsx	nucleoside-specific channel-forming protein	2.052355051	up	0.00145
PFLU2522		hypothetical protein	2.063485207	down	0.00613
PFLU2552	viscC	putative non-ribosomal peptide synthetase	2.088464499	up	0.00145
PFLU2553	viscB	putative non-ribosomal peptide synthetase	2.129778147	up	0.00106
PFLU2556		macrolide-specific ABC-type efflux carrier	2.228133678	up	0.000772
PFLU2596		hypothetical protein	2.549250019	down	0.000573
PFLU2617		putative ABC-type amino-acid transporter periplasmic solute-binding protein	2.070101211	down	0.00342
PFLU2757		hypothetical protein	2.049401083	down	0.00236
PFLU2792		putative molybdenum-pterin binding protein II	2.12064651	down	0.0017
PFLU2915		hypothetical protein	2.242607824	down	0.00362
PFLU2916		putative TetR family regulatory protein	2.488963	down	0.000687
PFLU3096		putative aminotransferase	2.173573767	down	0.000881
PFLU3188		glyoxalase/bleomycin resistance protein/dioxygenase superfamily protein	2.153156129	down	0.00507
PFLU3219		SyrP-like protein	2.179120149	down	0.000601
PFLU3315		phosphate ABC transporter ATP-binding protein	3.650459462	down	0.00354
PFLU3316		phosphate transporter permease	2.938114838	down	0.00635
PFLU3317		phosphate transporter permease	3.222665823	down	0.0092
PFLU3318		putative phosphate-binding periplasmic protein	6.50485614	down	0.00124
PFLU3323		putative amino acid permease membrane protein	6.798057079	up	0.000242
PFLU3324		hypothetical protein	6.564079762	up	0.000596
PFLU3325		putative rhizopine biosynthesis/dihydropicolinate synthase	7.021745205	up	0.000151
PFLU3326		putative fatty aldehyde dehydrogenase	5.737107754	up	0.000408
PFLU3327		putative D-amino acid dehydrogenase	5.193924427	up	0.00018
PFLU3372		hypothetical protein	2.110782623	up	0.00317
PFLU3525		putative acyl-CoA synthetase	2.666406393	up	0.0159
PFLU3526		acetyl-CoA acetyltransferase	2.319811821	up	0.0135
PFLU3527		putative dehydratase	2.314392567	up	0.0469
PFLU3528	fabG	3-ketoacyl-(acyl-carrier-protein) reductase	2.498208046	up	0.0214
PFLU3529		acyl-CoA dehydrogenase	7.878198147	up	0.00218
PFLU3532		putative fatty acid transporter membrane protein	10.29052544	up	0.00218
PFLU3533	praB	hypothetical protein	14.27752113	up	0.00137
PFLU3534	praA	hypothetical protein	12.75898743	up	0.00172
PFLU3535	alkB	putative alkane monooxygenase	9.506877899	up	0.00144
PFLU3536		putative family S33 unassigned peptidase	4.461949825	up	0.00413
PFLU3537		putative flavin-binding monooxygenase-like protein	5.785268784	up	0.00307
PFLU3538		putative short chain dehydrogenase	6.896756172	up	0.00284
PFLU3539		hypothetical protein	8.342073441	up	0.0022
PFLU3655		hypothetical protein	3.193084132	down	0.0000872
PFLU3656		hypothetical protein	2.825365382	down	0.000153
PFLU3657		hypothetical protein	2.232430151	down	0.00028
PFLU3658		putative extracellular polysaccharide biosynthesis protein	4.194696991	down	0.000853
PFLU3662		putative polysaccharide export protein	2.116813626	down	0.00235
PFLU3663		hypothetical protein	3.071488117	down	0.000496
PFLU3678		putative protein-tyrosine phosphatase	7.648847775	down	0.000175
PFLU3679		pseudo	2.537927129	down	0.00323
PFLU3745		putative ABC transporter ATP-binding protein	2.133700286	down	0.0347
PFLU3746		ATP-binding ABC transporter protein	2.059562032	down	0.043
PFLU3759		hypothetical protein	2.066760545	down	0.000178
PFLU3970		ornithine cyclodeaminase	2.045771054	down	0.000672
PFLU3981		putative GntR family transcriptional regulator	3.931177378	up	0.00195
PFLU3982		hypothetical protein	2.586524248	up	0.0102
PFLU3999		hypothetical protein	2.540869043	down	0.00917
PFLU4007	viscA	putative non-ribosomal peptide synthetase	2.383148193	up	0.000486
PFLU4154		putative inosine-uridine preferring nucleoside hydrolase	2.492651939	up	0.000908
PFLU4155		D-ribose pyranase	2.040247202	up	0.0104
PFLU4158		putative ribose transporter permease	2.098598719	up	0.00524
PFLU4161		putative L-asparaginase II	2.023008108	up	0.000881
PFLU4308		putative signaling-related membrane protein	2.207138106	down	0.000144
PFLU4332		long-chain-acyl-CoA synthetase	2.106747627	up	0.000253
PFLU4389	pvdS	extracytoplasmic-function sigma-70 factor	2.118157771	down	0.00491
PFLU4461		hypothetical protein	2.148850661	down	0.0142
PFLU4510		agmatinase	11.27319431	up	0.0000564
PFLU4511		hypothetical protein	8.390299797	up	0.000172
PFLU4512		putative transmembrane transport protein	3.863761187	up	0.000869
PFLU4513		putative sodium/solute symporter	5.941126823	up	0.000189
PFLU4536		hypothetical protein	7.46376786	down	0.013
PFLU4853		hypothetical protein	2.676332474	up	0.00511
PFLU4854		LysE-type translocator	2.098562479	up	0.0116
PFLU4889		putative amino acid transporter integral membrane protein	2.229698815	down	0.000575
PFLU4896		glycine cleavage system protein H	3.21936965	up	0.000598
PFLU5174		hypothetical protein	2.404265858	down	0.00087
PFLU5615	bioB	biotin synthase	2.368963964	down	0.000179
PFLU5618		hypothetical protein	3.313442663	down	0.00125
PFLU5656		putative oxidase	2.53435421	up	0.000333
PFLU5657		putative amine-transport related membrane protein	2.165042639	up	0.000852
PFLU5859		putative oxidoreductase	2.783037739	down	0.000288
PFLU6048		putative phosphate ABC transporter periplasmic substrate-binding protein	4.45596968	down	0.00216
PFLU6057	aspA	aspartate ammonia-lyase	3.497068405	up	0.000157
PFLU6061	aphA	acetylpolyamine aminohydrolase	3.407876968	up	0.00133
PFLU6062	potF	putrescine/polyamine-binding periplasmic protein	3.177605629	up	0.000801

Parent mass	Classes
970.591	T
1140.726	T
1148.68	T
1157.742	T
1158.77	T
1162.681	S
1162.709	ST
1163.767	ST
1164.85	T
1178.684	ST
1178.687	S
1180.69	T
1182.67	T
1184.77	T
1186.95	T

Parent mass	Classes
311.307	protozoa and interaction
325.322	Interaction
325.323	Interaction
451.461	protozoa and interaction
451.462	Interaction
477.478	Interaction
477.478	Interaction
477.478	Interaction
477.479	Interaction

Parent mass	Classes
752.518	T
766.536	T
768.495	T
770.517	S
772.594	T
782.513	T
782.514	T
782.56	T
796.531	AT
